# Single-cell RNA sequencing reveals the potential role of Postn(+) fibroblasts in promoting the progression of myocardial fibrosis after myocardial infarction

**DOI:** 10.1038/s41598-025-04990-6

**Published:** 2025-07-01

**Authors:** Wenyang Nie, Zhijie Zhao, Zhikai Xiahou, Jingwen Zhang, Yuhang Liu, Yong Wang, Zhen Wang

**Affiliations:** 1 First Clinical Medical College, Shandong University of Traditional Chinese Medicine, 16369 Jingshi Rd, Jinan, 250014 China; 2 Department of Plastic and Reconstructive Surgery, Shanghai ninth People’s Hospital, School of Medicine, Shanghai Jiao Tong University, 639 Zhi Zao Ju Rd, Shanghai, 200011 China; 3 China Institute of Sport and Health Science, Beijing Sport University, Beijing, 100084 China; 4 Department of Cardiovascular Diseases, Affiliated Hospital of Shandong University of Traditional Chinese Medicine, 16369 Jingshi Rd, Jinan, 250014 China

**Keywords:** Single-cell RNA sequencing, Myocardial infarction, Fibroblasts, Myocardial fibrosis, Postn, Bioinformatics, Cytological techniques, Sequencing, Biological techniques, Molecular biology, Stem cells, Biomarkers, Cardiology, Cell biology, Cell adhesion, Cell growth, Cell migration

## Abstract

Myocardial infarction (MI) is a life-threatening coronary artery-related pathology characterized by sudden cardiac death, often leading to cardiac fibrosis and heart failure (HF). Despite advances in emergency care and treatment measures such as percutaneous coronary intervention (PCI), the mortality rate due to HF following MI remains high, making it the leading cause of death in MI patients. While cardiac fibroblasts are known to be closely associated with the adverse outcomes of cardiac fibrosis and HF post-MI, the cellular landscape of fibroblasts after MI and their role in myocardial fibrosis and HF progression has not been fully explored. Our study identified a key, highly proliferative fibroblast subpopulation, named C1 Postn + Fibroblasts, which showed high myocardial fibrosis scores. C1 Postn + Fibroblasts were primarily located at the early stage of the pseudo-time trajectory and exhibited high stemness. These cells interact with EndoCs, ECs, and macrophages through the Cxcl12-Ackr3, Ptn-Ncl, and Mdk-Lrp1 signaling pathways, thereby influencing myocardial fibrosis progression. Additionally, Tead1 and Hdac2 were found to be key and highly active transcription factors in this subpopulation. In vitro experiments showed that knocking down Postn significantly decreased the activity of cardiac fibroblasts, inhibited their migration and adhesion capabilities, and induced apoptosis. This subpopulation may be more sensitive to post-MI adverse events, while other subpopulations may exhibit more inhibited responses. Stemness genes Ctnnb1 and Hifla, as well as oxidative phosphorylation and glutathione metabolism pathways, should be closely monitored in efforts to prevent myocardial fibrosis post-MI. The Cxcl12-Ackr3, Ptn-Ncl, and Mdk-Lrp1 pathways may represent potential routes to disrupt the key interactions between C1 Postn + Fibroblasts and EndoCs, ECs, and macrophages. Tead1 and Hdac2 may be potential targets for inhibiting myocardial fibrosis and preventing adverse outcomes of MI after further experimental verification. The gene Postn, expressed in C1 Postn + Fibroblasts, may contribute to the inhibition of abnormal fibroblast activation post-MI. These findings open new perspectives for the prevention and treatment of myocardial fibrosis after MI and the prevention of its progression to HF.

**Supplementary Information:**

The online version contains supplementary material available at 10.1038/s41598-025-04990-6.

## Introduction

Myocardial infarction (MI) is a life-threatening coronary artery pathology characterized by sudden cardiac death^1^. MI is a major cause of global mortality. Although it predominantly occurs in elderly patients, it increasingly threatens the lives of younger individuals due to differing and more numerous risk factors compared to the elderly^2^. Additionally, MI often presents with atypical signs and symptoms, making its early onset easy to overlook. Acute myocardial infarction (AMI) is a critical cause of morbidity and mortality in younger patients^3^often leading to heart failure (HF) and cardiac fibrosis^4,5^. Despite advancements in emergency care knowledge and percutaneous coronary intervention (PCI), the mortality rate due to HF post-MI remains high, being the primary cause of death among MI patients^6,7^.

The three most abundant cell types in the infarcted myocardium are cardiomyocytes, cardiac fibroblasts, and macrophages^8^. Post-MI, cardiac fibroblasts play a crucial role in the prognosis through their intrinsic actions and interactions with other cells. Cardiac fibroblasts are closely associated with adverse outcomes like cardiac fibrosis and HF post-MI. Ideally, macrophages and fibroblasts are the key cell types involved in healing post-MI, aiding in myocardial remodeling and fibrosis. The body maintains cardiac structural integrity post-MI by activating cardiac fibroblasts^9,10^. However, fibroblasts can proliferate abnormally, and excessively activated cardiac fibroblasts lead to fibrosis and HF^11^. Therefore, the transformation of fibroblasts into activated myofibroblasts is considered a critical step in cardiac fibrosis during MI^12^.

Studies indicate that specific pathway stimulations during MI can induce apoptosis in cardiomyocytes and fibrosis in cardiac fibroblasts^13^. Post-MI, macrophages also become activated and infiltrate necrotic myocardium as part of the inflammatory response^14^. However, excessive macrophage infiltration significantly reduces post-MI survival rates^15^. Notably, there are significant interactions between fibroblasts and macrophages. Macrophages stimulate cardiac fibroblasts to secrete IL-6, leading to fibrosis. Cardiac fibroblasts are activated by cytokines secreted by inflammatory cells, including macrophages, via certain signaling pathways, resulting in the production of type I and III collagen and eventual atrial fibrosis^16,17^. The interaction between cardiac fibroblasts and cardiomyocytes contributes to atrial fibrillation and plays a crucial role in myocardial fibrosis^18,19^. Furthermore, previous studies indicate a close relationship between cardiac fibroblasts and endothelial cells (ECs), highlighting the importance of fibroblasts in cardiovascular formation and potential diseases, including cardiomyopathy, MI, and myocardial fibrosis^20^.

Thus, cardiac fibroblasts may interact with cardiomyocytes, ECs, and macrophages through certain signaling pathways post-MI. However, comprehensive research on how fibroblasts influence disease progression and prognosis through these interactions post-MI is lacking. Unfortunately, no definitive research or therapy targeting fibroblasts post-MI has been universally recognized. The cellular landscape of fibroblasts and their role in myocardial fibrosis and HF post-MI are not fully understood. Additionally, it remains unclear whether the differential expression of stemness-related genes and transcription factors is associated with poor prognosis post-MI.

Therefore, in this study, we aim to visualize MI-associated fibroblasts by mining existing public data using Single-Cell RNA Sequencing (scRNA-seq). Specifically, we divided wild-type mice into two groups: one group underwent left thoracotomy followed by left anterior descending coronary artery ligation to establish the MI model (MI group), while the other group underwent sham ligation surgery without tightening the coronary artery (Sham group). Then, according to the heterogeneity between cells, the differences in cell stemness and development, and more importantly, the fibroblasts were scored for myocardial fibrosis, so as to screen and obtain the key fibroblast subpopulation we need, and to display the cell communication landscape between this subpopulation and other key cells, hoping to obtain the meaningful signaling pathways we expect, and use in vitro assays to verify the role of target genes. It provides some valuable insights for future strategies aimed at preventing the exacerbation of myocardial fibrosis in patients with myocardial infarction.

## Methods

### Data sources and processing

**Fig. 1:**
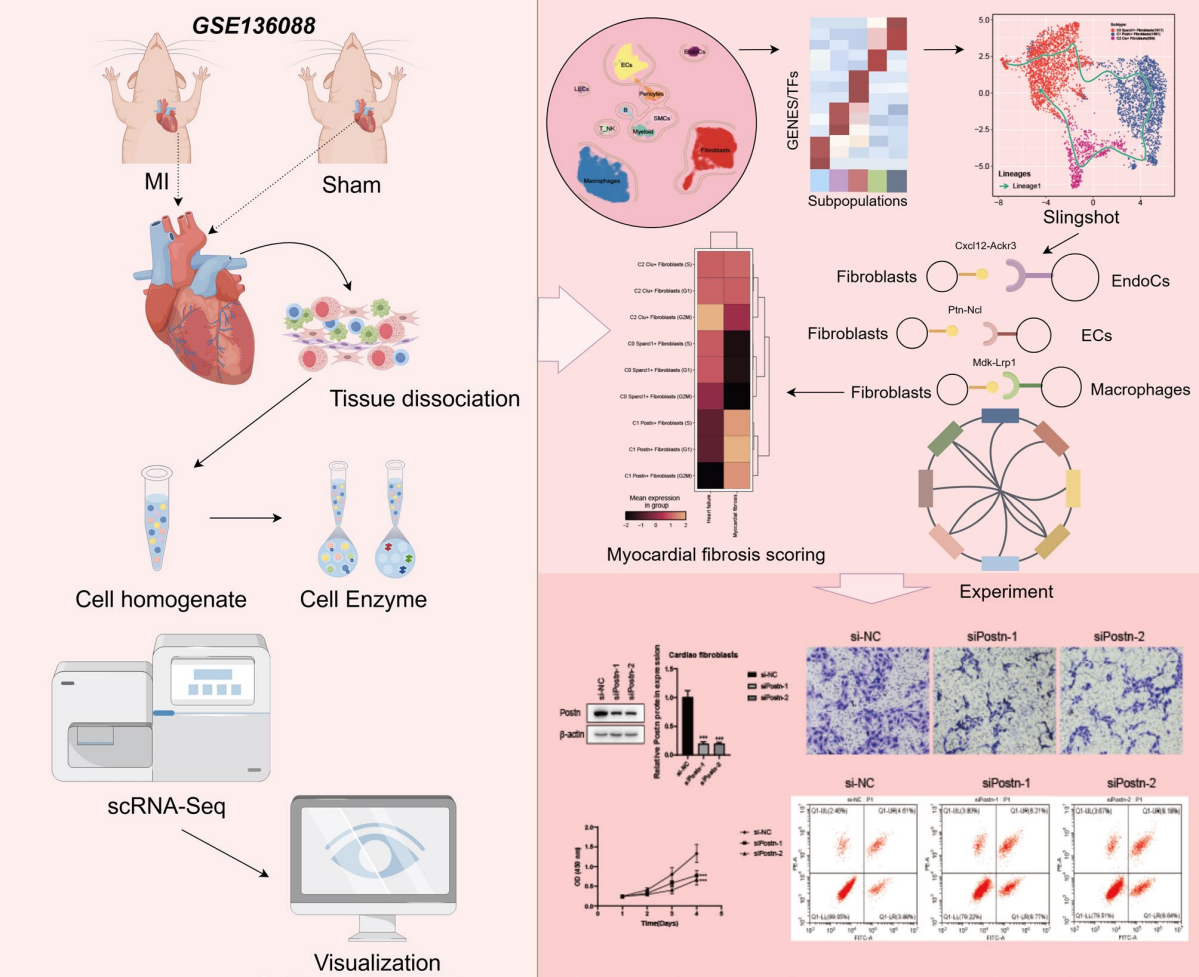
Research Overview. Myocardial fibrosis after myocardial infarction is closely related to cardiac fibroblasts. Single-cell RNA sequencing revealed that C1 Postn + Fibroblasts was a key subset that exhibite higher myocardial fibrosis scores. The potential effect of Postn was demonstrated by in vitro experiments.Pathways such as Cxcl12-Ackr3 may provide new directions for intervention.

The overall analysis workflow for this research was shown in Fig. 1. The scRNA-seq data of MI-associated fibroblasts used in this study were obtained from the GEO database (https://www.ncbi.nlm.nih.gov/geo/) under accession number GSE136088, specifically sample IDs GSM4040774 (MI group) and GSM4040775 (Sham group). Our study was reviewed and approved by the Ethics Committee of the Affiliated Hospital of Shandong University of Traditional Chinese Medicine (Designation: AF/SC-08/03.0). All authors confirm that all experiments were performed in accordance with the guidelines and regulations specified and that the ARRIVE guidelines were strictly adhered to. Specific details of the selected data are as follows: MI was modeled in C57BL6/J wild-type (WT) 8-to 10-week-old male mice (Jackson Laboratory) and confirmed by echocardiographic analysis. A left thoracotomy was performed through the fourth intercostal space, and the lungs were retracted to expose the heart. After opening the pericardium, the left anterior descending coronary artery was secured with a 7 − 0 silk suture approximately 2 mm below the margin of the left atrial appendage. Ligation was considered successful when the anterior wall of the left ventricle turned white. The lungs were inflated by increasing positive end-expiratory pressure and closed with a 6 − 0 suture layer in the open-chest position. Animals were maintained on a heating pad at 37 °C until recovery and 2 h after surgery. Another group of mice underwent sham ligation using a similar surgical procedure without tightening the suture around the coronary arteries. Mice with an estimated aortic coarctation pressure gradient below 40 mmHg were not included in the experiments. Hearts were collected 14 days after sham (*n* = 4, combined) or MI (*n* = 1).

The 10X Genomics data from each sample were processed using the Seurat package (v4.1.1) in R software (v4.1.3). Potential doublets were identified and removed using the DoubletFinder tool (v2.0.3)^21–23^and low-quality cells were filtered out. Quality control criteria were strictly applied, retaining only cells with 300 < nFeature < 4000 and 500 < nCount < 15,000 for downstream analysis. Additionally, mitochondrial gene expression was restricted to less than 20% of total counts, and the proportion of red blood cells was limited to below 5%. Furthermore, low-quality cells with fewer than 500 or more than 6000 identified genes were excluded. These rigorous thresholds ensured the inclusion of high-quality cells for subsequent analysis.

### Dimensionality reduction, clustering, and cell type identification in scRNA-seq analysis

Gene expression counts for each cell were first normalized to a scale of counts per 10,000 and subsequently log-transformed using the formula log(x + 1), where x denotes the normalized expression value. Following this, the top 2,000 highly variable genes (HVGs) were identified^24–26^. The expression matrix was then standardized, and principal component analysis (PCA) was performed based on the expression of these HVGs^27–30^. To address batch effects across samples, we applied the Harmony algorithm to the PCA embeddings.

To further minimize the influence of sample variability on downstream analyses, especially in trajectory inference using Monocle and Slingshot, we embedded cells in the Harmony-corrected low-dimensional space. Trajectory graphs were constructed using batch-adjusted principal components, and pseudotime progression was cross-validated across individual samples to ensure robustness. This approach effectively controlled for confounding factors associated with sample heterogeneity and improved the reliability of dynamic state inference. The top 30 principal components were subsequently selected for downstream dimensionality reduction and clustering analyses. The UMAP algorithm^31–33^ was then employed to visualize the clustering results in a two-dimensional space, laying the foundation for subsequent cell type identification^34,35^.

Cell type annotations were performed by referencing marker genes identified in the CellMarker database (http://xteam.xbio.top/CellMarker/) and relevant literature. These marker genes were used to label the derived cell clusters and assign specific cell types. Additionally, fibroblasts identified through this process were further reclustered to delineate distinct subpopulations. Differential marker genes specific to each fibroblast subcluster were used for their characterization and identification.

### Enrichment analysis and aucell

To identify differentially expressed genes (DEGs) for each cell type, we employed the FindMarkers function^35^ with default parameters based on the Wilcoxon rank-sum test. DEGs were selected from clusters where the log-fold change (logFC) exceeded 0.25 and were expressed in more than 25% of the cells within the cluster. To further elucidate the functional roles of these DEGs in each cell type, enrichment analyses were conducted using the clusterProfiler (v4.6.2) and SCP (v0.4.8) packages. Pathway analyses were performed using Gene Ontology (GO)^36–39^ terms and Gene Set Enrichment Analysis (GSEA) to uncover the biological processes and pathways associated with each cell type^40–43^.

Additionally, we applied the AUCell method to identify gene sets with activity signatures in scRNA-seq data^44^. AUCell calculates the “activity score” for a given gene set within each cell, providing insights into the functional activation states across individual cells. This approach allowed us to characterize cellular heterogeneity and gene set activity more comprehensively.

### Slingshot and CytoTRACE

To investigate the developmental and differentiation dynamics among fibroblast subpopulations following MI, we utilized the Slingshot package (v2.6.0)^45–47^ to construct developmental trajectories. This approach enabled us to infer the developmental stages and states of each fibroblast subpopulation. To validate the results and rank the differentiation states of all fibroblast subpopulations, we employed CytoTRACE^48,49^.

The functions getLineage and getCurves from the Slingshot package were used to infer differentiation trajectories for each subpopulation. Additionally, these tools facilitated the evaluation of temporal changes in the expression levels of differentially expressed genes along the inferred trajectories. This comprehensive analysis provided insights into the developmental progression and functional divergence of fibroblast subpopulations in the post-MI context.

### CytoTRACE2 analysis

We employed CytoTRACE2 to analyze the scRNA-seq data, aiming to predict the latent categories and absolute developmental potential of fibroblasts in both the MI and Sham groups. In CytoTRACE2, latent categories classify cells based on their developmental potential, while the predicted potential scores provide a continuous measure of developmental potency, ranging from 0 (fully differentiated) to 1 (pluripotent).

This method enables direct cross-dataset comparisons of developmental potential within an absolute framework, offering a robust approach to assess the cellular heterogeneity and differentiation states of fibroblasts across different conditions.

### Pseudotime trajectory of fibroblast subpopulations

To explore the differentiation dynamics of fibroblast subpopulations after MI, we utilized the Monocle package^34,50,51^ (v2.24.0) to construct pseudotime trajectories. This analysis, based on scRNA-seq data, enabled the reconstruction of fibroblast differentiation pathways in pseudotime, providing insights into the cellular transitions and progression occurring during the post-MI fibroblast differentiation process.

Monocle’s pseudotime framework facilitates the identification of key changes in cellular states along the differentiation trajectory, offering a deeper understanding of the developmental heterogeneity and functional evolution of fibroblast subpopulations.

### Cell-Cell communication

We employed the CellChat package (v1.6.1) to analyze and visualize intercellular interactions based on scRNA-seq data^52–55^. This analysis provided a comprehensive overview of communication networks among all identified cell types. Of particular importance, we focused on detailed visualizations of interactions between key fibroblast subpopulations and other critical cell types, including endocardial cells, endothelial cells, and macrophages. This allowed us to identify crucial signaling pathways underlying these interactions.

The CellChatDB.human database served as the reference for ligand-receptor interactions. To ensure statistical rigor, we applied a significance threshold of *P* < 0.05 when predicting intercellular communication between different cell types. This approach facilitated the identification of biologically meaningful signaling events in the myocardial infarction microenvironment.

### SCENIC analysis

SCENIC is a tool designed to reconstruct gene regulatory networks (GRNs) based on scRNA-seq data, with a particular focus on identifying stable cell states^56^. In this study, we applied the PySCENIC package (v0.10.0) in Python (v3.7) using default parameters to perform regulatory network reconstruction and assess transcription factor activity.

An AUCell matrix was generated to evaluate the enrichment of transcription factors and the activity of their corresponding regulators, providing insights into the regulatory landscape governing cellular states and transitions^50^. This approach enabled us to characterize the gene regulatory architecture of fibroblast subpopulations and their functional roles in the post-MI microenvironment.

### Cell culture

The cardiac fibroblasts cell line was sourced from the mouse cardiac fibroblasts of Procell in Wuhan, China. The cells were cultured in MEM culture medium supplemented with 10% fetal bovine serum, penicillin (100 U/mL), and streptomycin (0.1 mg/mL). Cultures were maintained in a standard incubator at 37 °C, 5% CO2, and saturated humidity. Cells in the logarithmic growth phase were harvested for subsequent experiments.

### SiRNA transfection

For the experimental procedure, cells were seeded onto 6-well plates at a density of 2 × 10^5 cells per well. After 24 h, the cells were transfected with siRNAs obtained from GenePharma (Shanghai, China) at a final concentration of 20 µM. Transfection was performed using RNAiMax (Life Technologies, ThermoFisher Distributor; Brendale QLD, Australia) following the manufacturer’s instructions. Cells were collected 24 h post-transfection for further analysis. The sequences of the siRNAs used in the experiment were as follows: siRNA1:AGCCUUGUAUGUAUGUUAU; siRNA2:GUGACAGUAUAACAGUAAA.

### RNA extraction and quantitative real-time PCR

To isolate total RNA, TRIzol reagent from Thermo Fisher Scientific (Waltham, MA, USA) was used following the manufacturer’s instructions. Subsequently, 500 ng of RNA was used for cDNA synthesis using the PrimeScript RT Reagent Kit from TaKaRa (Tokyo, Japan). For quantitative real-time PCR, the SYBR^®^ Premix Ex Taq™ from TaKaRa was utilized. PCR was conducted on the ABI V7 instrument from ABI (Indianapolis, IN, USA). The specific primers used for amplification were designed for the Postn gene as follows: Forward primer: 5’-AGGTCACCAAGGTCACCAAA-3’ and Reverse primer: 5’-TGTTGGCTTGCAACTTCCTC-3’.

### Western blotting analysis

The extraction of total cellular protein was processed by lysis buffer (1 M Tris-HCL, pH 6.8, 10% SDS and 80% glycerin). Bicinchoninic acid (BCA) kit was used for the determination of protein concentration follow the manufacturer’s instruction. Briefly, 10% SDS-PAGE gel was applied for separating the 30 µg total protein, and then proteins were electrophoretically transferred to polyvinylidene fluoride (PVDF) membranes (Millipore; Burlington, MA, USA). In the subsequent step, the membranes were washed three times with Tris buffered saline + Tween (TBST) and blocked thoroughly with 5% skim milk. Incubation with primary antibodies was performed overnight on blocked PVDF membranes at 4 °C. Secondary antibodies were applied for 2 h after primary antibody incubation. The detection of protein bands was verified with enhanced chemiluminescence kit (ECL; ThermoFisher Scientific, Waltham, MA, USA).

### Cell viability assay

Cell viability was evaluated using the Cell Counting Kit-8 (CCK-8) from DOJINDO (Kumamoto, Japan). Cells were seeded at 1 × 10^3 cells per well in 96-well plates and cultured overnight. A 100 µL detection reagent was added to each well and incubated for 1 h. Absorbance at 450 nm was measured daily over 4 days. Growth curves were plotted by correlating OD450 values with time to assess cell viability.

### Transwell migration and adhesion assay

A Transwell assay was performed to evaluate cell migration and adhesion. For the migration assay, 1 × 10^5 cells were harvested in 100µL of serum-free culture medium and added into the upper chamber, without Matrigel. Next, 600 µL of 30% fetal bovine serum medium was placed into the bottom compartment of the chamber as a source of chemo-attractant. After 24 h of culturing, the cells that crossed the inserts were fixed and strained with crystal-violet. Migrated cells were photographed and counted via an inverted microscope (100X magnification).

For the adhesion assay, transwell filters were pre-coated with 1% sterile gelatin (Sigma, Cat. #G-2500) and exposed to different treatments. After 2 h of culturing, the plates were gently washed with PBS to remove the non-adherent cells. The attached cells were then fixed with 4% formaldehyde and stained with Wright’s-Giemsa. Attached cells were photographed and counted using a microscope with 100X magnification.

### Flow cytometry for apoptosis analysis

To evaluate cellular apoptosis, the Annexin V-FITC/PI apoptosis detection kit from Sigma-Aldrich, Germany, was employed. Cells were incubated and washed with cold PBS, followed by suspension in a binding buffer containing Annexin V-FITC and PI stains. After incubation in the dark, the stained cells were analyzed using flow cytometry. Flow cytometry data was processed using FlowJo (v10.0.7) software to quantify the extent of apoptosis within the cell population.

### Statistical analysis

Statistical analysis was performed using R software (v4.3.0) and Python software (v4.2.0). Wilcoxon’s test and Pearson correlation coefficient were employed to assess the significance of differences between different groups. Significance levels were indicated as follows: *P < 0.05, **P < 0.01, ***P < 0.001, ****P < 0.0001. The term “ns” was used to denote non-significant differences between groups. These statistical tests and significance indicators were utilized to evaluate the statistical significance of the findings and provide confidence in the results.

## Results

### Annotation and functional insights into fibroblast subpopulations in MI and Sham groups

**Fig. 2:**
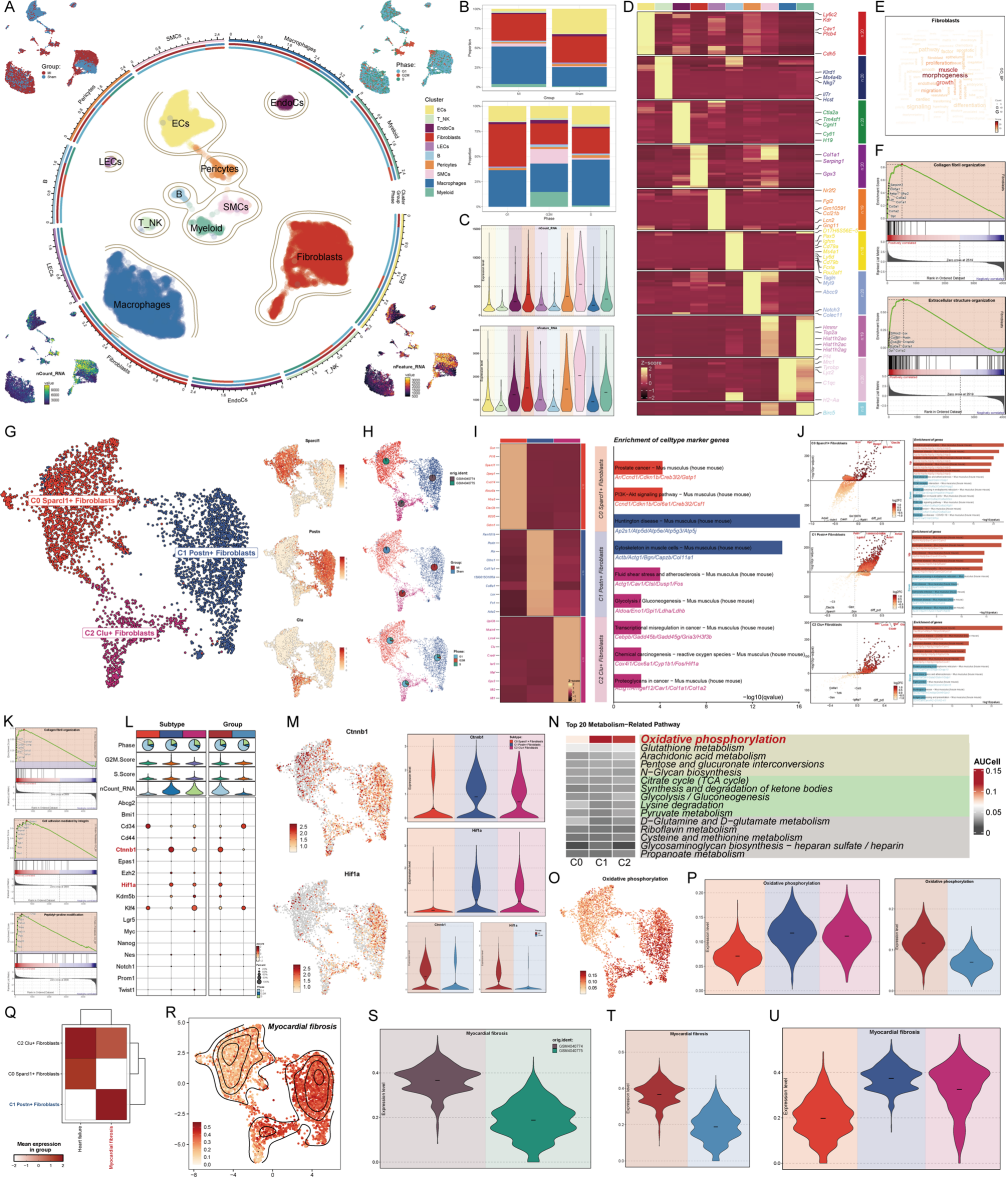
Characterization of Fibroblast Subpopulations in the MI and Sham Groups. (**A**) Differentially expressed genes were used to identify 10 distinct cell types from high-quality filtered cells in the MI and Sham groups. The distribution of cells from both groups across these cell types was depicted, along with their cell cycle states. Additionally, differences in nCount RNA and nFeature RNA across cell types were presented. (**B**) Bar plots illustrated the proportion of various cell types in the MI and Sham groups, as well as the composition of cell cycle states within each cell type. (**C**) Violin plots provided a comparative visualization of nCount RNA and nFeature RNA levels among different cell types. (**D**) Differentially expressed genes were identified for each of the 10 cell types. (**E**) The word cloud map displayed enriched terms related to fibroblasts. (**F**) GSEA results for fibroblasts were presented. (**G**) Based on differentially expressed marker genes, three fibroblast clusters were annotated as subpopulations and named Sparcl1+, Postn+, and Clu + Fibroblasts, according to their most distinct marker genes. (**H**) UMAP plots showed the sample origin and grouping of the three fibroblast subpopulations, along with the proportions of cells in different cell cycle states within each subpopulation (It is worth noting that in the data used in this study, the sample source GSM number corresponds to the group one by one: GSM4040774 corresponds to the MI group, and GSM4040775 corresponds to the Sham group). (**I**) Differentially expressed genes of the three fibroblast subpopulations were analyzed using GO-BP analysis. (**J**) Volcano plots highlighted the upregulated and downregulated genes in the three fibroblast subpopulations, with separate GO-BP analyses performed for each group. (**K**) GSEA results for C1 Postn + Fibroblasts were presented. (**L**) Bubble plots demonstrated differences in stemness gene expression among the subpopulations and between the groups. (**M**) UMAP and violin plots showed the expression levels of the stemness-related genes Ctnnb1 and Hif1a in the C1 subpopulation, comparing their rankings across subpopulations and groups. (**N–P**) The significant metabolic pathways for the three fibroblast subpopulations were analyzed using AUC values. The highest-scoring pathway for C1 Postn + Fibroblasts was oxidative phosphorylation, and its differences across subpopulations were visualized using UMAP and violin plots. Additionally, differences between the MI and Sham groups were highlighted. (**Q**) The heatmap compared heart failure and myocardial fibrosis scores across the three fibroblast subpopulations. (**R**) The UMAP plot displayed the distribution and density variations of myocardial fibrosis scores. (**S–U**) Violin plots compared myocardial fibrosis scores across different samples, groups, and subpopulations.

In GSE136088, although only one sample (*n* = 1) was included in the MI group, it was included in the subsequent analysis to initially explore the cellular profile of the post-MI heart. This sample was of high quality and showed good overall agreement with the sham group. Considering the exploratory nature of single-cell transcriptome studies, such designs have also been used in the previous literature^57–61^. Nonetheless, we are cautious about the interpretation of the results in this group. Subsequently, after batch correction of all cells in dataset GSE136088, we identified 13,658 high-quality cells and classified them into 26 clusters (Supplementary Fig. 1A). Using marker genes from existing literature, we characterized the expression profiles across these clusters (Supplementary Fig. 1B). Specifically, based on fibroblast-related markers (Supplementary Fig. 1C, D) and additional cell-specific markers, we annotated these clusters as fibroblasts, macrophages, T-NK cells, B cells, smooth muscle cells (SMCs), endothelial cells (ECs), myeloid cells, lymphatic endothelial cells (LECs), endocardial cells (EndoCs), and pericytes (Fig. 2A). The majority of macrophages, T-NK cells, B cells, myeloid cells, SMCs, and fibroblasts originated from the MI group, while a smaller proportion came from the Sham group. Myeloid cells, LECs, and EndoCs were approximately evenly distributed between the two groups, whereas ECs were predominantly derived from the Sham group, with only a minor contribution from the MI group. Notably, SMCs and myeloid cells were mostly in the G2/M phase, along with a subset of fibroblasts, the majority of which were from the MI group. Most other cells were found in the G1 phase. Additionally, fibroblasts exhibited higher RNA counts and gene expression levels compared to other cell types. Further analysis revealed that macrophages and fibroblasts were the most abundant cell types in the MI group, while ECs, fibroblasts, and macrophages dominated in the Sham group. Among cells in the G2/M phase, macrophages, fibroblasts, ECs, and SMCs were highly represented, whereas cells in the G1 phase were primarily fibroblasts and macrophages (Fig. 2B). Consistent with UMAP results, fibroblasts had RNA counts and gene numbers second only to SMCs (Fig. 2C). We hypothesize that fibroblasts exhibit significant proliferative activity. Metabolic pathway analysis indicated that oxidative phosphorylation and glutathione metabolism were the most active pathways in fibroblasts. UMAP and violin plots highlighted the distinctly higher activity of these pathways in fibroblasts compared to other cell types (Supplementary Fig. 1E-G). To further explore fibroblasts, we conducted enrichment analyses. Heatmap visualization of differentially expressed genes (DEGs) across all cells identified Col1a1, Serping1, and Gpx3 as specifically expressed in fibroblasts (Fig. 2D). Enrichment of DEG sets revealed terms highly associated with fibroblasts, such as morphogenesis, muscle, and growth, as shown in the word cloud (Fig. 2E). GSEA demonstrated upregulated genes enriched in collagen fibril organization and extracellular structure organization pathways (Fig. 2F).

To validate the active growth and proliferative capacity of fibroblasts, we explored their stemness and the differential expression of stemness-associated genes. Bubble plot analysis revealed elevated expression of stemness genes, including Cd34, Kdm5b, Twist1, and Hif1a, in fibroblasts (Supplementary Fig. 1H), which was corroborated by UMAP results showing their higher expression in fibroblasts compared to other cell types (Supplementary Fig. 1I). Violin plots provided a clearer visualization of these differences (Supplementary Fig. 1J). After batch correction, 4,377 fibroblasts from the MI and Sham groups were categorized into three clusters based on specific marker genes: C0 Sparcl1 + Fibroblasts, C1 Postn + Fibroblasts, and C2 Clu + Fibroblasts (Fig. 2G). C2 Clu + Fibroblasts were predominantly derived from GSM4040774 (MI group), with a subset in the G2/M phase, while C0 Sparcl1 + Fibroblasts originated mainly from GSM4040775 (Sham group) and a small proportion from the MI group. Notably, C1 Postn + Fibroblasts were almost exclusively from the MI group and largely in the G1 phase (Fig. 2H). Differential expression analysis revealed that C1 Postn + Fibroblasts specifically expressed genes such as Acta2, Fn1, Lox, Postn, Ptn, Col11a1, Cthrc1, Fam101b, and Col8a1, with Ptn being particularly notable (Fig. 2I). GO-BP analysis showed enrichment in pathways like Huntington’s disease and cytoskeleton organization in muscle cells, while volcano plots highlighted enrichment in pathways such as Parkinson’s disease, Huntington’s disease, prion disease, Alzheimer’s disease, and diabetic cardiomyopathy (Fig. 2J). GSEA analysis revealed significant upregulation of pathways related to collagen fibril organization, cell adhesion mediated by integrins, and peptidyl-proline modification in C1 Postn + Fibroblasts (Fig. 2K). Stemness-related gene expression showed that C1 Postn + Fibroblasts highly expressed genes such as Ctnnb1 and Hif1a, particularly in the MI group (Fig. 2L-M). Metabolic pathway analysis demonstrated that oxidative phosphorylation in C1 Postn + Fibroblasts had the highest AUC scores, both within the subpopulation and compared to other fibroblast subpopulations, as confirmed by UMAP and violin plots (Fig. 2N-P).

To further elucidate the potential associations between key fibroblast subpopulations and myocardial fibrosis or heart failure, we performed scoring analyses for these conditions across all subpopulations. A heatmap provided detailed scoring information for myocardial fibrosis and heart failure across subpopulations in different cell-cycle phases (Supplementary Fig. 1K). C1 Postn + Fibroblasts exhibited consistently high scores for myocardial fibrosis across all cell-cycle phases, significantly surpassing other subpopulations (Fig. 2Q). In contrast, heart failure scores were higher in C2 Clu + Fibroblasts, while C1 Postn + Fibroblasts showed relatively lower scores, even below those of C0 Sparcl1 + Fibroblasts, which were predominantly derived from the Sham group. The density distribution and comparative results of heart failure scores across subpopulations were consistent with the heatmap findings (Supplementary Fig. 1L, M). For myocardial fibrosis scores, C1 Postn + Fibroblasts displayed the highest density (Fig. 2R). To validate the accuracy of myocardial fibrosis scoring, we also analyzed scores at the sample and group levels, confirming significantly higher scores in GSM4040774 and the MI group, aligning with expected results (Fig. 2S, T). Violin plots further illustrated that C1 Postn + Fibroblasts prominently exhibited myocardial fibrosis characteristics (Fig. 2U).

### Pseudotime landscape and stem cell differentiation potential of fibroblast subpopulations

**Fig. 3:**
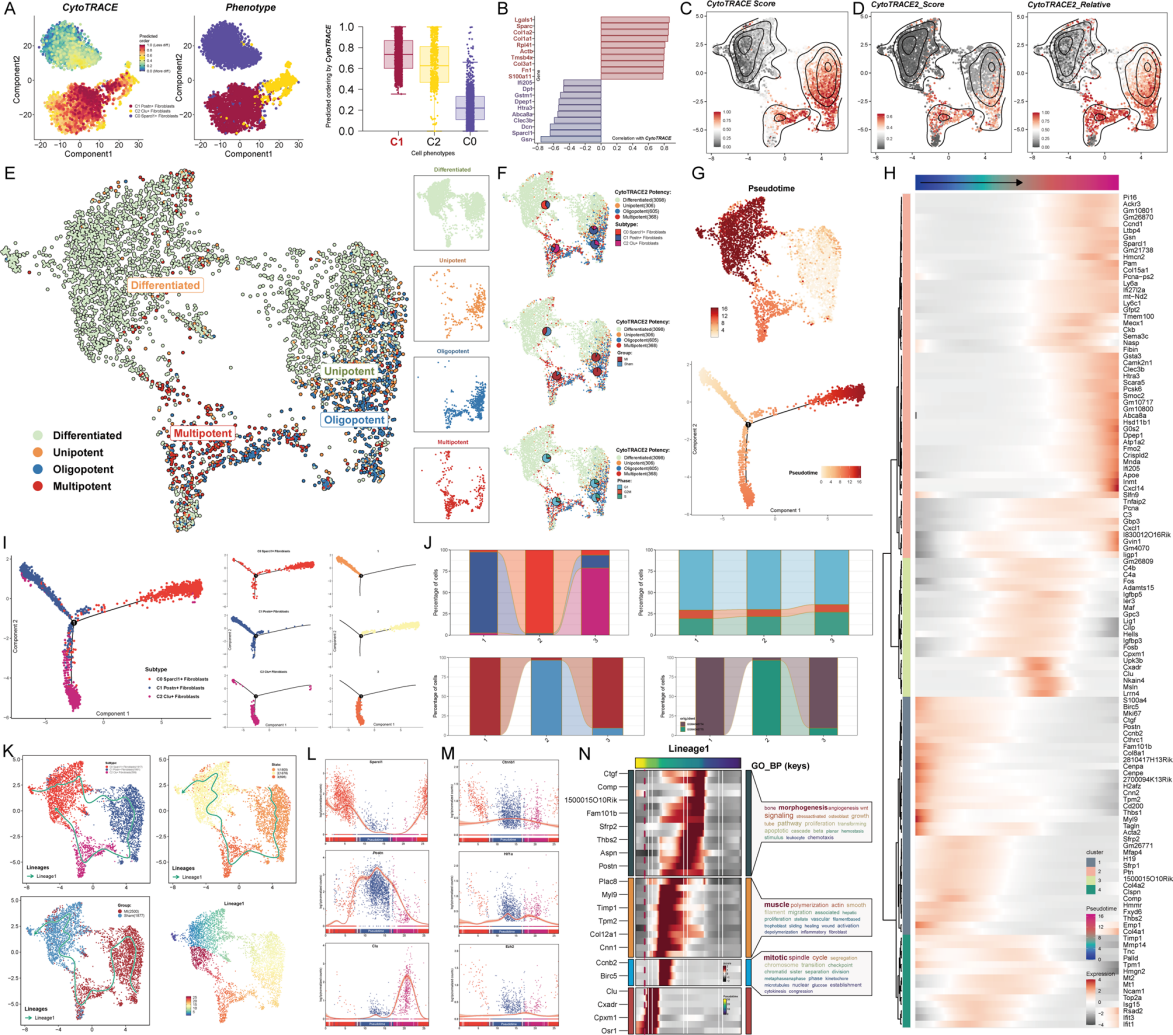
Pseudotime Analysis Reveals the Heterogeneity of Fibroblast Subpopulations. (**A**) CytoTRACE analysis highlighted differences in differentiation states among the three fibroblast subpopulations. (**B**) Genes positively and negatively correlated with CytoTRACE scores were identified. (**C**) The UMAP plot depicted the distribution of CytoTRACE scores across subpopulations, showing variations in score magnitude and density. (**D**) The UMAP plots displayed CytoTRACE2 scores and CytoTRACE2 relative scores, along with their distribution density across subpopulations. (**E**) The UMAP plots illustrated differences in stemness potential among the fibroblast subpopulations. (**F**) The proportions of each fibroblast subpopulation within high-stemness cells were analyzed, alongside the proportions of cells from different groups and cell cycle states within these high-stemness populations. (**G**) Pseudotime trajectories constructed using Monocle were visualized on UMAP plots, where the progression was indicated by a gradient of colors. The trajectory developed from the top-left to the right, featuring a bifurcation point. (**H**) Expression of subpopulation-specific marker genes along the pseudotime trajectory was analyzed. (**I**) Subpopulation distributions along the pseudotime trajectory were mapped based on marker gene expression. The trajectory was divided into three states (state1-state3) according to the bifurcation point. (**J**) Bar plots illustrated the composition of subpopulations, cell cycle states, groups, and samples within state1, state2, and state3. (**K**) Slingshot analysis revealed a pseudotime developmental trajectory (Lineage1), visualized on UMAP plots. The trajectory’s progression across fibroblast subpopulations was as follows: C0 Sparcl1 + Fibroblasts → C1 Postn + Fibroblasts → C2 Clu + Fibroblasts → C0 Sparcl1 + Fibroblasts. Lineage1 trajectories were also mapped across different groups and states. (**L**) Temporal changes in the expression of subpopulation-specific marker genes along Lineage1 were analyzed. (**M**) Temporal expression patterns of the stemness-related genes Ctnnb1, Hif1a, and Ezh2 along Lineage1 were presented. (**N**) Differentially expressed genes across all subpopulations showed distinct temporal patterns along Lineage1. These genes were categorized into four gene clusters based on their expression timing, followed by enrichment analysis for each cluster.

We employed CytoTRACE analysis to evaluate the differentiation potential of fibroblast subpopulations. C1 Postn + Fibroblasts exhibited lower specificity, as indicated by CytoTRACE scores, and box plots demonstrated that this subpopulation had the lowest differentiation degree (Fig. 3A). Genes correlated with CytoTRACE scores were identified, with positive correlations observed for Lgals1, Sparc, Col1a2, Col1a1, Rpl41, Actb, Tmsb4x, Col3a1, Fn1, and S100a11, and negative correlations for Ifi205, Dpt, Gstm1, Dpep1, Htra3, Abca8a, Clec3b, Dcn, Sparcl1, and Gsn (Fig. 3B). C1 Postn + Fibroblasts and C2 Clu + Fibroblasts showed significantly higher CytoTRACE and CytoTRACE2 scores, including relative CytoTRACE2 values (Fig. 3C, D). UMAP visualization from CytoTRACE2 analysis revealed that all three subpopulations contained differentiated cells. Unipotent stem cells were primarily located in C1 Postn + Fibroblasts, oligopotent stem cells were mainly found in C1 Postn + Fibroblasts and C2 Clu + Fibroblasts, while multipotent stem cells were predominantly distributed in C2 Clu + Fibroblasts, with a smaller proportion in C1 Postn + Fibroblasts (Fig. 3E, F). Unipotent, oligopotent, and multipotent stem cells were primarily derived from the MI group, with only a minor fraction of multipotent stem cells originating from the Sham group. Across all differentiation capacities, most stem cells were in the G1 phase, followed by the G2/M phase (Fig. 3F).

Using Monocle, we constructed a pseudotime trajectory to reveal the developmental progression of fibroblast subpopulations. The trajectory began with C1 Postn + Fibroblasts at the early stage, followed by C2 Clu + Fibroblasts, and culminated with C0 Sparcl1 + Fibroblasts at the late stage, as visualized through UMAP (Fig. 3G). The trajectory unfolded from left to right and featured a branching point. Differential gene expression along the trajectory was visualized, revealing distinct expression patterns at various pseudotime stages (Fig. 3H). By mapping the subpopulations based on the temporal expression patterns of their marker genes, C1 Postn + Fibroblasts were predominantly situated at the early stage, C2 Clu + Fibroblasts at the mid-stage, and C0 Sparcl1 + Fibroblasts at the late stage. The trajectory was divided into three states based on the branching point: State1 (early stage), State2 (mid-stage), and State3 (late stage) (Fig. 3I). State1 was primarily composed of C1 Postn + Fibroblasts, exclusively derived from the MI group and sample GSM4040774. State2 consisted largely of C2 Clu + Fibroblasts, mainly from the Sham group (sample GSM4040775), while State3 contained approximately 75% C0 Sparcl1 + Fibroblasts, along with some C1 Postn + Fibroblasts and C2 Clu + Fibroblasts, mostly originating from the MI group. Across all states, the majority of cells were in the G1 phase, followed by the G2/M phase (Fig. 3J). Using Slingshot, we further constructed a developmental trajectory for all fibroblast subpopulations, identifying a single lineage, Lineage1, in which the sequential order was C0 Sparcl1 + Fibroblasts → C1 Postn + Fibroblasts → C2 Clu + Fibroblasts → C0 Sparcl1 + Fibroblasts. The directional progression of Lineage1, reconstructed based on different states and groups, was consistent (Fig. 3K). To validate the positional accuracy of subpopulations along Lineage1, we visualized the expression of their defining genes. Sparcl1 was prominently expressed at the early stage, Postn at the mid-stage, and Clu at the late stage (Fig. 3L). Additionally, stemness-related genes highly expressed in C1 Postn + Fibroblasts, such as Ctnnb1, Hif1a, and Ezh2, were mapped onto the pseudotime trajectory. Both Ctnnb1 and Hif1a exhibited significant expression during the early and mid-stages of Lineage1, while Ezh2, though expressed at lower levels overall, showed relatively higher expression during the C1 Postn + Fibroblasts stage (Fig. 3M).

Finally, we visualized temporal expression differences of various genes along Lineage1 using a heatmap, clustering genes with similar expression patterns. Enrichment analysis of these clusters revealed that genes primarily expressed in the early to mid-stages, such as Postn and Sfrp2, were associated with terms like morphogenesis, bone, angiogenesis, Wnt signaling, and growth (Fig. 3N).

### Key pathways involved in crosstalk between C1 Postn + Fibroblasts and Endocs, Ecs, and macrophages

**Fig. 4:**
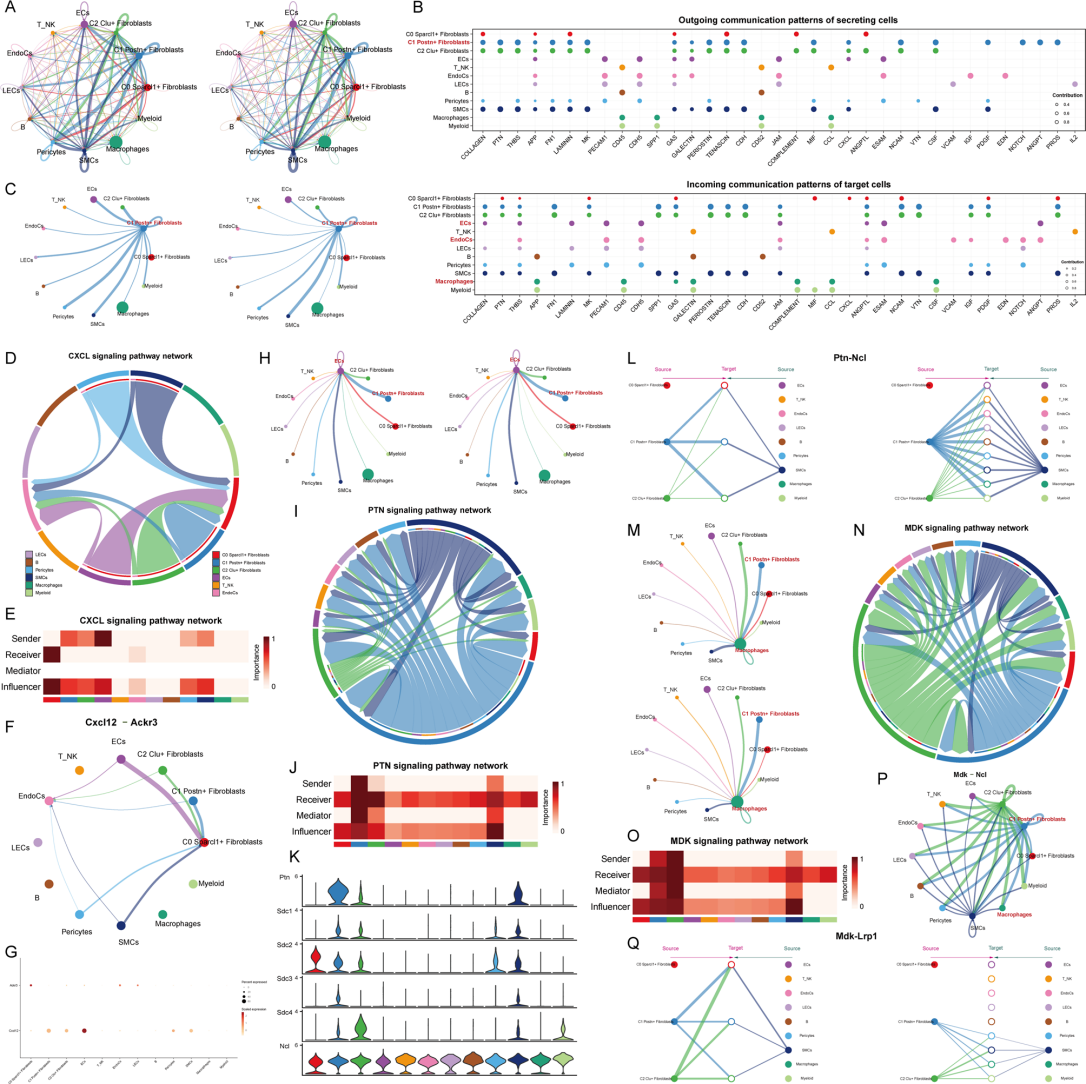
Crosstalk Between the C1 Subpopulation and EndoCs, ECs, and Macrophages. (**A**) Circos plots displayed the intensity and number of interactions between all fibroblast subpopulations and other cell types. (**B**) The expression levels of ligand-receptor pairs when all cell types acted as signal senders (top) and as signal receivers (bottom). (**C**) Circos plots highlighted the interaction intensity and count when C1 Postn + Fibroblasts acted as signal senders and interacted with other cell types. (**D**) Chord diagrams illustrated the interactions of all cell types within the CXCL signaling pathway. (**E**) The heatmap quantified the influence of each cell type in the CXCL signaling pathway as signal senders, receivers, or intermediaries. (**F**) The circos plot represented the interactions among all cell types within the Cxcl12-Ackr3 signaling pathway. (**G**) The differential expression of ligand Cxcl12 and receptor Ackr3 across all cell types. (**H**) Circos plots displayed the interaction intensity and count when ECs acted as signal receivers from other cell types. (**I**) Chord diagrams illustrated the interactions among all cell types in the PTN signaling pathway. (**J**) The heatmap evaluated the influence scores of all cell types acting as different roles within the PTN signaling pathway. (**K**) Expression differences of PTN-related receptor proteins across cell types in the PTN signaling pathway were analyzed. (**L**) All cell types interact autocrine and paracrine in the Ptn-Ncl signaling pathway. (**M**) Circos plots displayed the interaction intensity and count when macrophages acted as signal receivers from other cell types. (**N**) Chord diagrams represented the interactions of all cell types within the MDK signaling pathway. (**O**) The heatmaps scored the influence of all cell types in different roles within the MDK signaling pathway. (**P**) The circos plot showed the interactions among all cell types in the Mdk-Ncl signaling pathway. (**Q**) Autocrine and paracrine roles in Mdk-Lrp1 signaling in all cell types.

To investigate the crosstalk between C1 Postn + Fibroblasts and MI-associated EndoCs, ECs, and macrophages, we first visualized the interaction strength and number among all cell types using circle diagrams (Fig. 4A). This overview revealed the complexity of intercellular communications in both MI and Sham groups. A bubble plot further detailed the differential expression of receptor proteins in signal-receiving cells and ligand proteins in signal-sending cells across various cell types (Fig. 4B). Focusing on the key C1 Postn + Fibroblasts subpopulation, we examined its specific interactions with EndoCs, ECs, and macrophages, given their relevance to MI prognosis. A dedicated chord diagram highlighted the number and strength of interactions between C1 Postn + Fibroblasts and other cells, showing notably strong interactions with ECs (Fig. 4C).

We then visualized interactions between C1 Postn + Fibroblasts and EndoCs. The chord diagram in Fig. 4D detailed signals targeting EndoCs, including contributions from other cells such as C0 Sparcl1 + Fibroblasts, confirming the interaction between C1 Postn + Fibroblasts and EndoCs. Interestingly, analysis of the CXCL signaling pathway network revealed that C1 Postn + Fibroblasts acted as a significant sender, while EndoCs were primary receivers within this pathway (Fig. 4E). Further investigation identified the Cxcl12-Ackr3 axis as a specific ligand-receptor pair mediating the interaction between C1 Postn + Fibroblasts and EndoCs (Fig. 4F). A bubble plot validated this finding, showing that the ligand Cxcl12 was expressed in C1 Postn + Fibroblasts, while the receptor Ackr3 was expressed in EndoCs (Fig. 4G). This suggests that the Cxcl12-Ackr3 axis plays a critical role in the communication between these cell types during MI.

Using the same approach, we continued to visualize the interactions of C1 Postn + Fibroblasts with ECs. First, we analyzed the interaction strength and number between ECs (as the receiver) and other cell types (Fig. 4H). In the PTN signaling network, C1 Postn + Fibroblasts played a prominent role, actively influencing ECs (Fig. 4I). In the heatmap, C1 Postn + Fibroblasts showed a strong contribution as a sender, while ECs were significant as receivers (Fig. 4J). Based on these observations, we hypothesized that there might be specific ligand-receptor pairs in the PTN signaling pathway that mediate the interaction between C1 Postn + Fibroblasts and ECs. This was further validated by the violin plot, which showed higher expression of Ptn in C1 Postn + Fibroblasts, and significant expression of Ncl in ECs (Fig. 4K). Moreover, in the Ptn-Ncl signaling pathway, C1 Postn + Fibroblasts exerted paracrine effects on ECs, with notable interaction strength (Fig. 4L).

Next, we turned our attention to macrophages, which we had previously identified as significant. Again, we displayed the interaction strength and number between all cells and macrophages (Fig. 4M). As observed earlier, C1 Postn + Fibroblasts exhibited high interaction intensity and quantity with macrophages, especially in terms of strength. Within the MDK signaling network, we discovered that only SMCs, C1 Postn + Fibroblasts, and C2 Clu + Fibroblasts interacted with macrophages (Fig. 4N). C1 Postn + Fibroblasts contributed significantly as the sender, while macrophages also showed considerable contribution as receivers (Fig. 4O). Further analysis of the Mdk-Ncl ligand-receptor pair revealed that C1 Postn + Fibroblasts played a dominant role in influencing macrophages (Fig. 4P). Additionally, in the Mdk-Lrp1 signaling pathway, C1 Postn + Fibroblasts exerted paracrine effects on macrophages with a strong interaction intensity (Fig. 4Q). Moreover, we believed that the mutual crosstalk between the two in Mdk-Lrp1 is of greater significance for exploration.

### Gene regulatory network analysis of fibroblast subpopulations

**Fig. 5:**
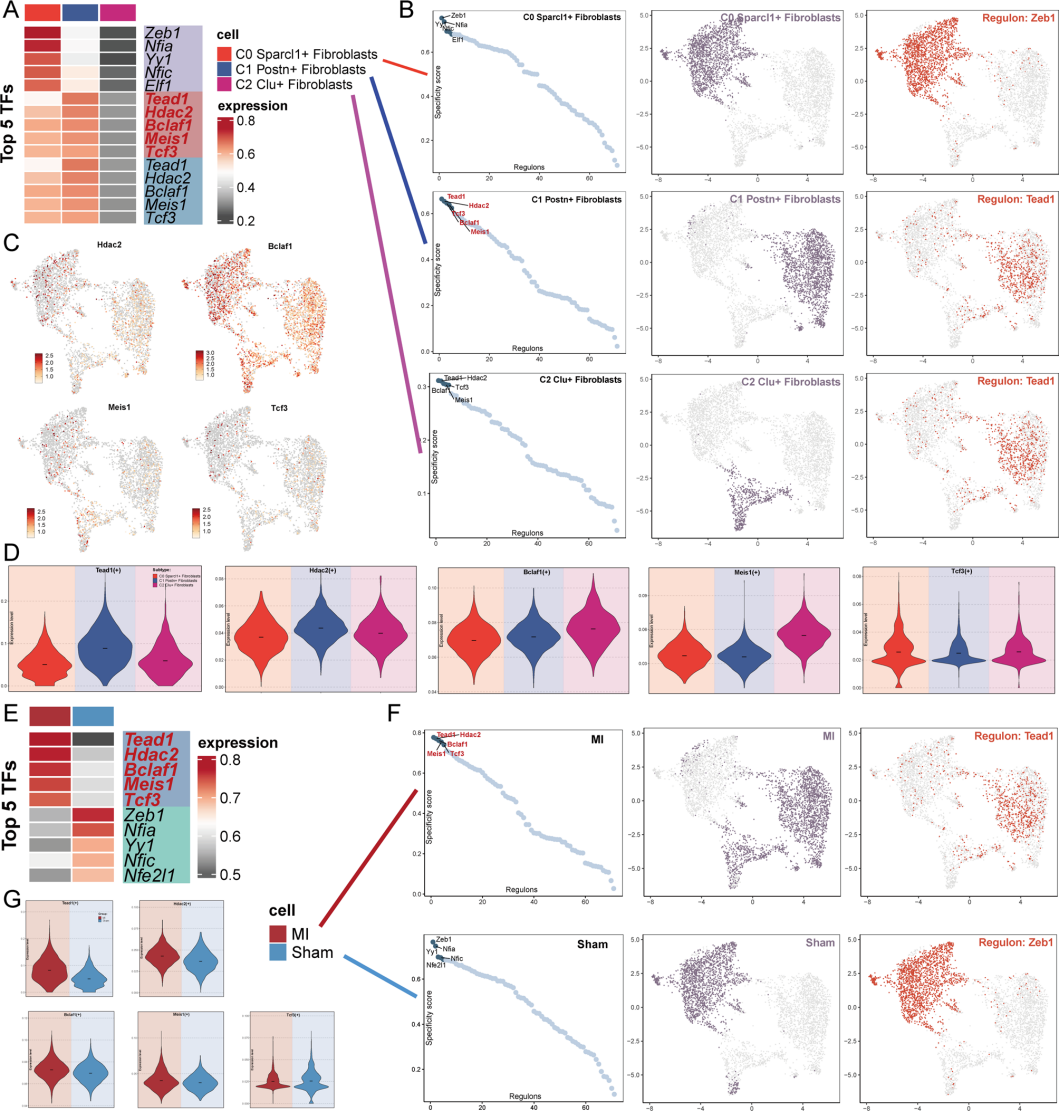
Gene Regulatory Networks of Fibroblast Subpopulations in the MI and Sham Groups. (**A**) The heatmap displayed the differential expression of the top five TFs in the three fibroblast subpopulations. (**B**) Fibroblast subpopulations were highlighted as light purple dots in the UMAP plot (middle). The ranking of regulatory factors within subpopulations based on their Regulator Specificity Scores (RSS) was shown (left). The binary Regulator Activity Scores (RAS) of the highest regulatory factors (normalized by Z-score with a cutoff of 2.5, converted to 0 or 1) were mapped as red dots on the UMAP plot (right). (**C**) UMAP plots illustrated the expression patterns of Hdac2, Bclaf1, Tcf3, and Meis1 across all subpopulations, excluding Tead1. (**D**) Violin plots compared the AUC values of five TFs (Tead1, Hdac2, Bclaf1, Tcf3, and Meis1), ranking their activity across fibroblast subpopulations. (**E**) The heatmap highlighted the differential expression of the top five TFs between the MI and Sham groups. (**F**) The MI and Sham group distributions were highlighted as light purple dots in the UMAP plot (middle). The ranking of regulatory factors in the two groups based on their RSS scores was displayed (left). Binary RAS scores of the top regulatory factors, mapped as red dots, illustrated their distribution across the UMAP plot (right). (**G**) Violin plots depicted the differences in AUC values for the five TFs (Tead1, Hdac2, Bclaf1, Tcf3, and Meis1) between the MI and Sham groups.

To identify the core transcription factors (TFs) detectable in fibroblasts from both the MI and Sham groups, we employed PySCENIC for analysis. First, we visualized the Top 5 TFs across all fibroblast subpopulations (Fig. 5A). The Top 5 highly expressed TFs in C1 Postn + Fibroblasts were Tead1, Hdac2, Bclaf1, Meis1, and Tcf3. Next, we ranked the regulatory factors of each fibroblast subpopulation according to their Regulatory Specificity Scores (RSS) and highlighted specific subpopulations in the UMAP with light purple dots. Additionally, we mapped the highest regulatory factor for each fibroblast subpopulation based on their binaryized Regulatory Activity Scores (RAS), displaying their distribution with red dots, which aligned with the heatmap results (Fig. 5B). We then mapped the expression distribution of the four TFs (Hdac2, Bclaf1, Meis1, Tcf3) (excluding Tead1) onto the UMAP, confirming their expression in C1 Postn + Fibroblasts (Fig. 5C). We further displayed the differences in the AUC values of the TFs (Tead1, Hdac2, Bclaf1, Meis1, Tcf3) across the three fibroblast subpopulations using a violin plot (Fig. 5D). We observed that C1 Postn + Fibroblasts exhibited the highest AUC values for Tead1 and Hdac2 compared to the other subpopulations. Additionally, we identified differential expression of TFs between the MI and Sham groups. The Top 5 TFs in the MI group were Tead1, Hdac2, Bclaf1, Meis1, and Tcf3, which mirrored the Top 5 TFs in C1 Postn + Fibroblasts (Fig. 5E). We then ranked the regulatory factors of both groups based on their RSS and highlighted the respective group in the UMAP with light purple dots. The mapping of the highest regulatory factors from both groups onto the UMAP with red dots showed consistency with the heatmap (Fig. 5F). Finally, we compared the AUC values of these 5 TFs between the MI and Sham groups using a violin plot, revealing that the AUC values for Tead1, Hdac2, Bclaf1, and Meis1 were higher in the MI group than in the Sham group, while the difference for Tcf3 was not significant (Fig. 5G).

### Identification and visualization of TF regulatory modules in fibroblast subpopulations

**Fig. 6:**
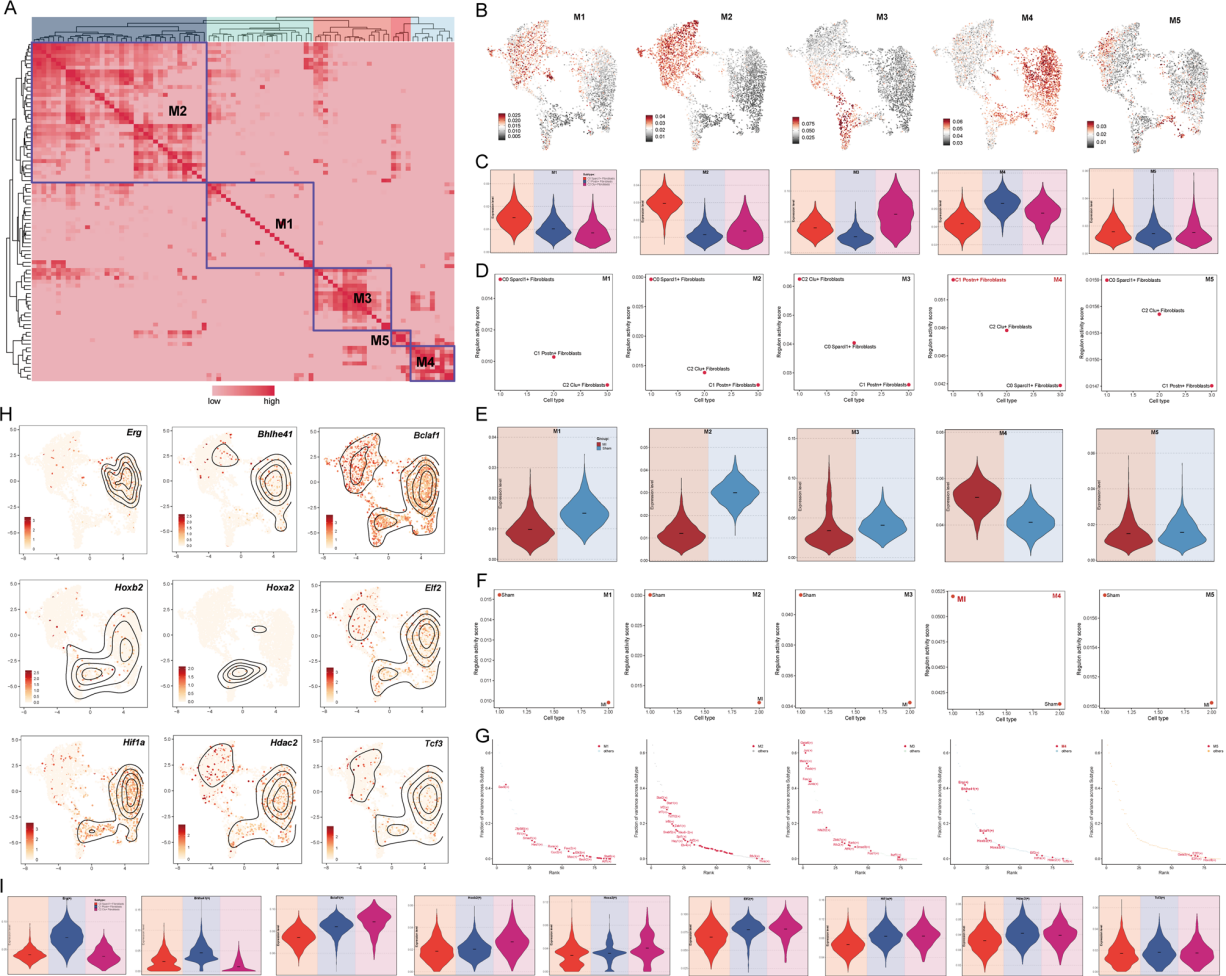
Identification of TFs Regulatory Modules in Fibroblasts. (**A**) The heatmap illustrated the regulatory modules identified across all fibroblast subpopulations in the MI and Sham groups using PySCENIC. Modules were determined based on the similarity of regulatory rules and AUCell scores, leading to the identification of five regulatory submodules (M1–M5) based on rule similarity. (**B**) UMAP plots visualized the distribution of the five regulatory submodules (M1–M5) across all fibroblast subpopulations. (**C**) Violin plots displayed the proportions of fibroblast subpopulations within each module (M1–M5). (**D**) Rankings of regulatory activity scores for the three fibroblast subpopulations were shown within each module (M1–M5). (**E**) Violin plots compared the proportions of cells from the MI and Sham groups within each of the five regulatory submodules. (**F**) Regulatory activity scores for cells from the MI and Sham groups were shown for each module (M1–M5). (**G**) The top-ranking TFs with the highest activity in the five regulatory submodules were identified. (**H**) Intensity and density plots depicted the activity levels of the nine top-ranking TFs within module M4. (**I**) Violin plots compared the activity levels of the nine TFs among the three fibroblast subpopulations.

Initially, we used PySCENIC identification rules and the Connectivity Specificity Index (CSI) matrix to discover the regulatory modules in fibroblast subpopulations. Based on the AUCell scores, the regulatory modules were divided into five main modules (M1, M2, M3, M4, M5) (Fig. 6A). We then mapped the average activity score of each module onto the UMAP plot, revealing that each module predominantly occupied different fibroblast subpopulations (Fig. 6B). From the UMAP and violin plots, we observed that most of the cells in M1 and M2 were C0 Sparcl1 + Fibroblasts, while M3 was mainly comprised of C2 Clu + Fibroblasts. M4 had the highest proportion of C1 Postn + Fibroblasts, and M5 showed no significant differences among the subpopulations with generally low activity (Fig. 6C).

Next, we ranked all subpopulations based on their regulon activity scores for modules M1 to M5. As shown in Fig. 6D, the ranking was as follows:

- In M1, the order was C0 Sparcl1 + Fibroblasts > C1 Postn + Fibroblasts > C2 Clu + Fibroblasts.
- In M2, the order was C0 Sparcl1 + Fibroblasts > C2 Clu + Fibroblasts > C1 Postn + Fibroblasts.
- In M3, the order was C2 Clu + Fibroblasts > C0 Sparcl1 + Fibroblasts > C1 Postn + Fibroblasts.
- In M4, the order was C1 Postn + Fibroblasts > C2 Clu + Fibroblasts > C0 Sparcl1 + Fibroblasts.
- In M5, the order was C0 Sparcl1 + Fibroblasts > C2 Clu + Fibroblasts > C1 Postn + Fibroblasts.

Therefore, we deduced that the characteristics of M4 might somewhat represent the traits of C1 Postn + Fibroblasts. To further understand the source of cells in each module, the violin plot indicated that the number of cells from the Sham group was significantly higher in M1, M2, and M3 compared to the MI group. Conversely, M4 had a higher number of cells from the MI group than from the Sham group, while M5 showed no significant difference, with the Sham group being slightly higher than the MI group (Fig. 6E).

Following this, we ranked the regulon activity scores for cells derived from the MI group and Sham group in each module (M1 to M5). In M1, M2, M3, and M5, the Sham group had higher scores than the MI group, while in M4, the MI group had higher scores than the Sham group (Fig. 6F).

We also ranked the fraction of variance in TFs across subtypes for modules M1 to M5, shown in Fig. 6G. In M4, the top-ranked TFs were: Erg, Bhlhe41, Bclaf1, Hoxb2, Hoxa2, Elf2, Hif1a, Hdac2, and Tcf3. We visualized the expression of these 9 TFs and found that Erg, Bhlhe41, Bclaf1, Hoxb2, Elf2, Hif1a, Hdac2, and Tcf3 were expressed at higher densities in C1 Postn + Fibroblasts compared to other subpopulations (Fig. 6H). The violin plot confirmed that Erg, Bhlhe41, and Hdac2 exhibited higher expression levels in C1 Postn + Fibroblasts than in other subpopulations (Fig. 6I).

### Postn significantly affects the proliferation, migration, adhesion, and apoptosis of cardiac fibroblasts

**Fig. 7:**
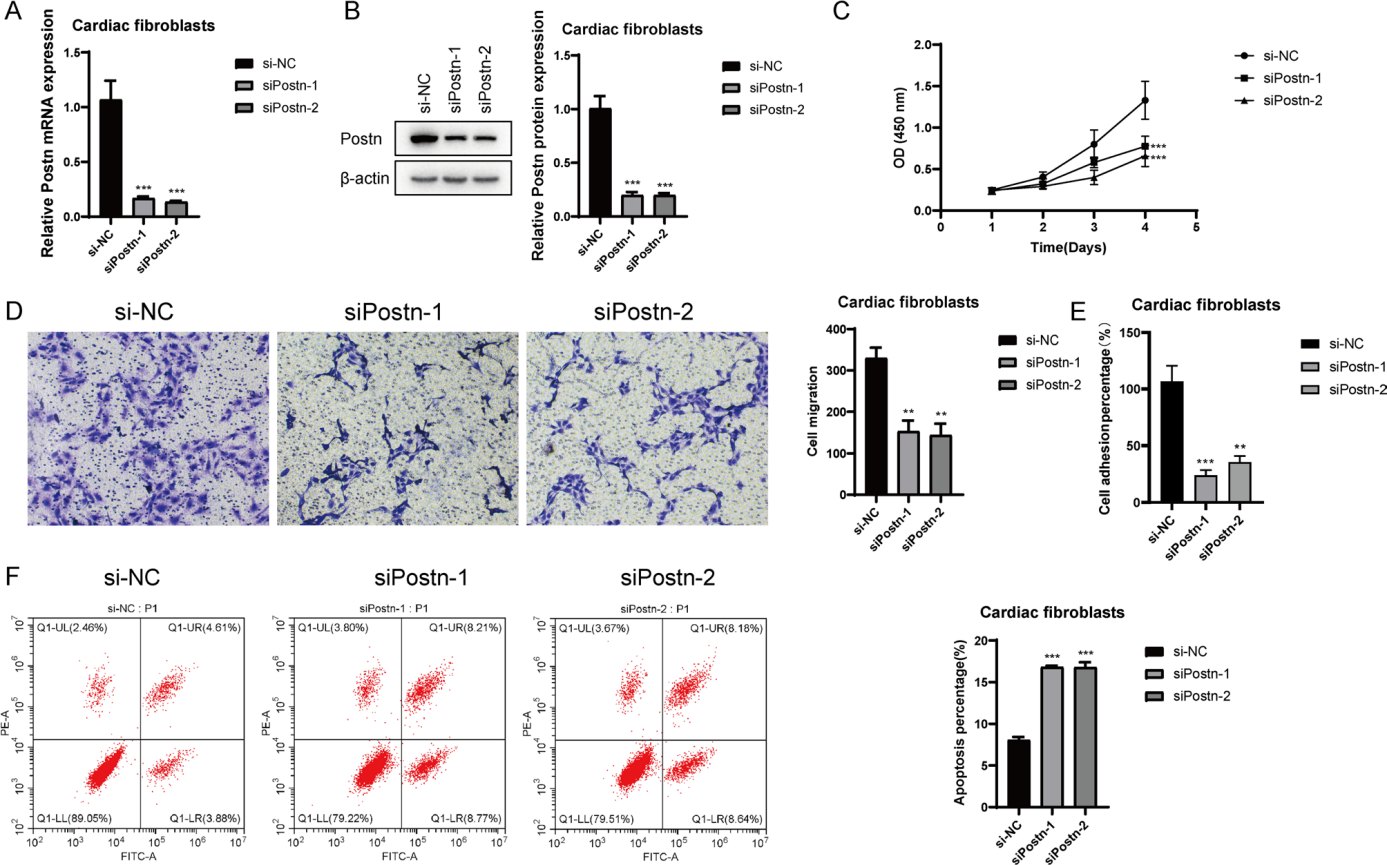
In Vitro Experiments on Postn Knockdown in Cardiac Fibroblasts. (**A**) Relative expression of Postn mRNA in cardiac fibroblasts with si-Postn knockdown. (**B**) Relative expression of Postn protein in cardiac fibroblasts with si-Postn knockdown. (**C**) CCK-8 assay demonstrated a significant decrease in cell viability following Postn knockdown. (**D**) Transwell migration assays showed that Postn knockdown significantly reduced the migration ability of cardiac fibroblasts. (**E**) Adhesion assays revealed a significant reduction in cell adhesion ability following Postn knockdown. (**F**) Annexin V-FITC/PI double staining flow cytometry showed that Postn knockdown significantly induced apoptosis in cardiac fibroblasts compared to the NC group.

In the final step of our study, to further elucidate the impact of Postn, the key gene associated with the C1 Postn + Fibroblasts subpopulation, on the cellular behaviors of cardiac fibroblasts, we conducted in vitro experiments. To maintain consistency with our previous data sources, we selected a mouse cardiac fibroblast cell line for this experiment. First, we measured Postn expression levels 24 h after transfection using qRT-PCR to determine the efficacy of siRNA-mediated Postn knockdown in cardiac fibroblasts (Fig. 7A). Next, we verified the relative expression of Postn protein after knockdown, and indeed, the Postn protein expression was significantly reduced (Fig. 7B). Additionally, the CCK8 assay confirmed that the knockdown of Postn significantly decreased the cell viability of cardiac fibroblasts (*P* < 0.001) (Fig. 7C). Transwell migration assays showed that Postn knockdown significantly reduced the migration capacity of cardiac fibroblasts (Fig. 7D). Moreover, as shown in Fig. 7E, Postn knockdown also markedly decreased the cell adhesion ability of cardiac fibroblasts. Finally, we performed Annexin V-FITC/PI staining and flow cytometry analysis to assess cell apoptosis. Knockdown of Postn increased the proportion of apoptotic cardiac fibroblasts (Fig. 7F). To ensure the accuracy and consistency of the results, all tests were performed using the same cardiac fibroblast cell line, and data are presented as the mean ± standard deviation from independent experiments.

## Disscusion

MI is a cardiovascular disease with high morbidity and mortality, leading to various pathophysiological changes in the heart, including ischemia/reperfusion injury, inflammation, fibrosis, and ventricular remodeling, ultimately resulting in heart failure^62^. MI has become a major public health threat, jeopardizing global population health and well-being^63^. Despite improvements in early survival rates and the delay in heart failure progression through treatment strategies like PCI, these therapies are limited in their ability to prevent or reverse fibrosis and adverse cardiac remodeling^64,65^. Furthermore, clinical translation of therapeutic strategies targeting signaling pathways with broad functions in inflammation and fibrosis has not been successful^66^. Therefore, we aim to identify potential pathways that could block myocardial fibrosis after MI using public data.

First, we observed that the proportion of fibroblasts was higher in the MI group, and these fibroblasts were predominantly in the G1 phase. Moreover, we found that oxidative phosphorylation and glutathione metabolism in fibroblasts were particularly active. Oxidative phosphorylation can promote cardiac fibroblast activation and contribute to heart fibrosis, potentially leading to the adverse consequences of heart failure^67^. Similarly, mitochondrial glutathione (mGSH) is a crucial molecule for maintaining mitochondrial redox homeostasis and serves as an effective indicator for studying oxidative stress related to myocardial fibrosis. Therefore, fluctuations in GSH levels offer new perspectives for the diagnosis and treatment of myocardial fibrosis^68–70^. This supports the potential link between the fibroblasts we are focusing on and the development of myocardial fibrosis after MI.

Subsequently, among the fibroblast subgroups we identified, the C1 Postn + Fibroblasts, named after Postn, drew our attention. Studies have shown that Postn expression increases in fibroblasts and muscle cells, particularly upregulating in myocardial fibrosis after MI^71,72^. Furthermore, it has been suggested that Postn + cells are precursors of cardiac fibrosis-associated myofibroblasts^73^. Notably, the oxidative phosphorylation activity in C1 Postn + Fibroblasts was the highest among all the subgroups, and their differentially expressed genes include Acta2, Fn1, Lox, Postn, Ptn, Col11a1, Cthrc1, Fam101b, and Col8a1. These genes are primarily enriched in pathways related to diabetic cardiomyopathy and peptidyl-proline modification. Additionally, fibroblasts are closely linked to mesenchymal cells. While fibroblasts are generally considered the main precursor cells for myofibroblasts^74,75^mesenchymal cells can also differentiate into pro-fibrotic myofibroblasts^75^. These mesenchymal cells are present in various organs, including the adult heart, and notably, after MI, they can differentiate into myofibroblasts to influence fibrosis^77–79^. Moreover, the upregulated genes in this subgroup, as indicated by GSEA, suggest that it may be closely associated with collagen fibril organization and cell adhesion mediated by integrins—both of which are implicated in cellular-level changes during myocardial fibrosis. Therefore, the C1 Postn + Fibroblasts are likely related to myocardial fibrosis following MI.

To better validate the selection of our key subgroup, we utilized the trajectories constructed by Monocle and Slingshot. In the trajectory built by Monocle, C1 Postn + Fibroblasts were positioned in the early and intermediate stages, suggesting that most of the fibroblasts in the MI group were also located in the initial phase. Additionally, Slingshot’s lineage analysis (Lineage1) also placed C1 Postn + Fibroblasts in the early developmental stages. Further analysis revealed that Ptn and Sfrp1 were highly expressed in C1 Postn + Fibroblasts. The role of Ptn has been shown to potentially promote angiogenesis and cardiac remodeling after MI^80,81^while Sfrp1 is a potential biomarker for MI^82,83^. The high expression of both Ptn and Sfrp1 was predominantly observed in the MI group, with little to no expression in the Sham group. This further supports that most of the fibroblasts in C1 Postn + Fibroblasts are activated after MI. Moreover, CytoTRACE analysis indicated that C1 Postn + Fibroblasts had a relatively low differentiation state, which suggests that a significant portion of these cells may be in the early stages of development and differentiation, with active cell proliferation. The results from CytoTRACE2 further revealed that C1 Postn + Fibroblasts contained a higher proportion of unipotent and oligopotent stem cells. Unipotent stem cells can differentiate into a specific cell type and only develop into a single cell lineage^84,85^. This highlights the specific differentiation potential of C1 Postn + Fibroblasts, where they can give rise to a narrow range of cell types, reinforcing their unique role in the context of MI-related fibrosis.

In addition, since we analyzed the stemness of C1 Postn + Fibroblasts, we found that the stemness-related genes Ctnnb1 and Hif1a are both highly expressed in these cells. Studies have shown that Ctnnb1 plays a dual role in cardiac development and is crucial for the asymmetry of the heart^86,87^. Additionally, Hif1a activity is known to be associated with fibrosis in vascular remodeling^88^. The key subgroup, C1 Postn + Fibroblasts, has the highest expression of Ctnnb1, suggesting that this gene may play a significant role in the progression of myocardial fibrosis after MI. To further investigate, we mapped Ctnnb1 expression onto the pseudotime trajectory, and the results showed that Ctnnb1 is indeed highly expressed in the early stages of Lineage1. Based on these findings, we hypothesize that Ctnnb1 may contribute to the detrimental effects of cardiac fibrosis post-MI, potentially aggravating the progression of myocardial fibrosis after injury.

Based on the above findings, the key subgroup C1 Postn + Fibroblasts appears to be in a phase of heightened activity after MI, which suggests that interactions between these cells and others may also be highly active. Previous studies have found that macrophages, through specific signaling pathways and phenotypic transitions, promote the transition from myocardial ischemia to cardiac fibrosis^89^. Additionally, fibroblasts and endothelial cells can transdifferentiate, and post-MI myocardial fibrosis is closely associated with endothelial-to-mesenchymal transition (EndMT)^90,91^which can exacerbate myocardial fibrosis and worsen heart dysfunction^92^. To explore these interactions, we visualized the potential interactions between C1 Postn + Fibroblasts, EndoCs, ECs, and macrophages and identified representative ligand-receptor pairs. One key finding was the Cxcl12-Ackr3 pathway, which was identified as having a high potential for cell communication. More excitingly, recent research by Rebeca et al. found that high expression of Ackr3 (also known as Cxcr7) mediates cell migration and invasion during endocardial buffering formation^92^. This suggests that C1 Postn + Fibroblasts may use the Cxcl12-Ackr3 pathway to promote excessive formation and expansion of the endocardium.

Furthermore, as mentioned earlier, EndMT can be induced and exacerbated post-MI, leading to increased cardiac fibrosis. The generation and proliferation of ECs are enhanced, while their apoptosis is reduced. This suggests that certain key regulators may drive ECs toward EndMT, thereby exacerbating myocardial fibrosis^94^. In our cell communication analysis, we identified a prominent Ptn-Ncl pathway. Both Ptn and Ncl have been shown to promote EC proliferation and migration, and studies indicate that blocking or downregulating the function of Ncl on ECs can suppress migration and capillary-tube formation, leading to EC apoptosis^95–97^. Therefore, the Ptn-Ncl pathway may serve as a potential mechanism to prevent EC proliferation and their transition to EndMT. Additionally, Ncl could be a key target for blocking excessive EC proliferation, which in turn could help mitigate the progression of cardiac fibrosis. This insight provides a new potential avenue for therapeutic intervention in post-MI myocardial fibrosis.

Finally, we identified the Mdk-Lrp1 pathway as a key receptor-ligand interaction between C1 Postn + Fibroblasts and macrophages, which drew our attention due to previous studies highlighting that the Mdk-Lrp1 interaction can promote M2 macrophage polarization^98^. Macrophage polarization, particularly M2 polarization, plays a crucial role in the progression of heart failure and cardiac fibrosis following ischemia/reperfusion (IR) injury^99^. Moreover, research has directly shown that M2 macrophage polarization promotes cardiac fibrosis^100^. Thus, the interaction between Mdk and Lrp1 may influence the polarization of macrophages to the M2 phenotype. “However, we recognize that it is important to highlight the temporal dynamics of macrophage polarization, particularly the transition from M1 to M2 macrophages after MI.” Although M2 macrophages can support tissue repair in the early stages of MI, if their activity persists, it may lead to excessive fibrosis, which in turn promotes the occurrence of pathological remodeling. The M1 to M2 polarization transition is a key process in the healing response after MI. Initially, M1-type macrophages dominate the inflammatory phase and promote tissue damage and inflammatory responses. As the healing process progresses, M2-type macrophages gradually become dominant, helping to repair tissue and eliminate inflammation^101^. However, if the M2 phenotype persists after the repair phase, it may shift from a beneficial repair role to a pathologic contribution that exacerbates fibrosis and hinders normal tissue remodeling. Therefore, we further emphasize that although M2-type macrophages play a crucial role in early repair after MI, their persistent activation may lead to excessive fibrosis and promote later stages of pathological remodeling. This dual property of M2 macrophages highlights the complexity of cardiac immune regulation and also emphasizes the importance of precise modulation of the timing of macrophage polarization in future therapeutic strategies for fibrosis intervention and promotion of normal cardiac repair. So we preliminarily thought that the longer the time after infarction, the more M2 macrophage polarization, the longer the duration, and the greater the possibility of excessive fibrosis. Then the greater the possibility that Mdk-Lrp1 signaling could be a target for intervention to block the progression of this disease.

The fibroblast-specific signaling pathways revealed above may provide new ideas for clinical intervention after myocardial infarction. At present, the main treatment methods for MI include early reperfusion therapy and anti-fibrosis strategies for cardiac remodeling. However, existing methods still have certain limitations in improving long-term cardiac function. The Cxcl12-Ackr3 axis we found is not only closely associated with specific fibroblast subsets in this study, but also has been shown to play a protective role in angiogenesis and tissue repair after myocardial infarction^102^suggesting that it may be a potential target for promoting tissue remodeling after reperfusion. In addition, Ptn-Ncl and Mdk-Lrp1 pathways also play important roles in cardiac fibrosis and matrix remodeling^103,104^. By regulating these pathways, it is expected to achieve precise control of fibroblast activity, thereby reducing adverse myocardial remodeling and promoting orderly repair. Taken together, our findings provide a theoretical basis for exploring therapeutic strategies targeting the fibroblast signaling pathway after MI, which may be complementary to existing treatments in the future, thereby increasing the possibility of improving the long-term prognosis of patients.

After identifying the key receptor-ligand pairs (Cxcl12-Ackr3, Ptn-Ncl, and Mdk-Lrp1), we turned our attention to the highly expressed TFs in C1 Postn + Fibroblasts. Using PySCENIC analysis and visualization, we confirmed that the TFs with the highest expression in C1 Postn + Fibroblasts are Tead1 and Hdac2. Previous studies, such as those by Tsika et al., have demonstrated that elevated Tead1 levels can induce cardiac remodeling features associated with cardiomyopathy and heart failure in mouse models^105^. Similarly, inhibition of Hdac proteins has been shown to reduce inflammation, hypertrophy, and fibrosis in the heart^106^. Thus, Tead1 and Hdac2 could serve as key TFs for inhibiting cardiac fibrosis. We then categorized the regulatory modules for all fibroblast subpopulations, where the M4 module exhibited characteristics that could, to some extent, represent the features of C1 Postn + Fibroblasts. In M4, the variance scores^106^ for Erg and Bhlhe41 were higher, indicating greater variability in their expression, and C1 Postn + Fibroblasts, the main constituent of M4, were relatively depleted. Meanwhile, the scores for Tcf3, Hdac2, and Hif1a were lower in M4. The consistent results for Tcf3 and Hdac2 reinforce their roles, while the lack of direct studies linking Tcf3 with post-MI cardiac fibrosis led us to further verify the connections of Hif1a and Hdac2 with cardiac fibrosis.

We would like to outline that several clinical and preclinical anti-fibrosis strategies are currently under investigation in addition to the targets explored in this study. These strategies include the use of angiotensin-converting enzyme inhibitors (ACEI), angiotensin II receptor antagonists (ARBs), and mineralocorticoid receptor antagonists (MRAs), treatments that have shown varying degrees of effectiveness in reducing fibrosis in a variety of organs, including the heart^108–110^. In addition, strategies targeting TGF-β signaling, a key mediator of fibrosis, are also being actively advanced, with multiple clinical trials testing TGF-β receptor or downstream signaling inhibitors^111,112^. Targeted therapy of Tead1 and Hdac2 could complement existing anti-fibrotic strategies by providing alternative mechanisms to regulate fibrotic responses. Specifically, inhibition of Tead1 and Hdac2 may enhance the efficacy of current therapies by targeting gene expression programs and epigenetic modifications that drive fibrosis. Given the current lack of effective therapies to reverse fibrosis, especially in advanced stages, targeting Tead1/Hdac2 may provide a new strategy for fibrosis remission in patients who do not respond well to conventional therapies^113^. In addition, inhibition of Tead1 and Hdac2 could be an alternative strategy for those patients who have a poor response to current antifibrotic therapies. This strategy may be particularly important in those patients who continue to develop fibrosis despite conventional therapy, offering new hope for improving the prognosis of patients with advanced fibrotic disease. Thus, the potential role of Tead1/Hdac2 as a complementary or alternative strategy in anti-fibrosis therapy expands the therapeutic field and highlights the need to continue investigating this strategy in future clinical trials^114^.

To directly demonstrate the characterization differences among the fibroblast subpopulations, we scored each subpopulation based on relevant pathways and specific genes. Notably, in the subsequent myocardial fibrosis scoring, C1 Postn + Fibroblasts had the highest score, which strongly supports our initial hypothesis. Furthermore, this score was also the highest in the MI group, providing further evidence that myocardial fibrosis is a major early adverse outcome following MI. In contrast, the heart failure scores did not show significant differences across the subpopulations, with C1 Postn + Fibroblasts scoring relatively low. We hypothesize that this might be because C1 Postn + Fibroblasts are in a relatively active phase, likely consisting of highly activated fibroblasts, with myocardial fibrosis being their primary characteristic. Heart failure, on the other hand, is the ultimate adverse outcome, primarily occurring in the later stages of disease progression. This temporal discrepancy between the early activation of C1 Postn + Fibroblasts and the later onset of heart failure may explain the lower heart failure score for this subpopulation. This could be a positive sign for preventing the malignant progression of secondary diseases post-MI.

Taken together, the C1 Postn + Fibroblasts we identified in this study exhibit distinct profibrotic gene expression signatures, higher proliferative potential in the early stages, and active cellular communication with endothelial and immune cells after MI, suggesting that this subset may play a key role during adverse cardiac remodeling. Although the correlation between C1 subgroup and cardiac function indicators (such as infarct size and ejection fraction) has not been directly evaluated in this study, it has been documented that the expansion of Postn + fibroblasts is closely related to increased collagen deposition and adverse cardiac remodeling after myocardial infarction^115^. In addition, the enrichment degree of Postn + cells was also found to be negatively correlated with the decrease of left ventricular ejection fraction (LVEF)^116^suggesting its potential as a predictive marker for the deterioration of cardiac function after myocardial infarction. These results further highlight the potential value of the possible existence of the fibroblast subsets we identified in the clinic and provide a rationale for subsequent exploration of them as therapeutic targets.

Finally, we align with the view that the abnormal activation of cardiac fibroblasts is the primary cause and hallmark of cardiac fibrosis^117^. Therefore, we conducted in vitro experiments on the key subpopulation, C1 Postn + Fibroblasts, and confirmed that the expression of the gene Postn significantly enhances the proliferation, migration, and adhesion of cardiac fibroblasts, while notably inhibiting their apoptosis. This suggests that Postn may be the key driver behind the abnormal activation of cardiac fibroblasts. Consequently, targeting the inhibition of fibroblast activation could potentially serve as a therapeutic strategy to prevent cardiac fibrosis following MI. However, the broader cell-to-cell interactions suggested by CellChat, such as fibroblast-macrophage or fibroblast-endothelial signaling pathways, have not been experimentally tested. To address this issue, we plan to validate these interactions by co-culture or conditioned medium experiments in future studies. These experiments will provide direct experimental data on the signaling pathways between fibroblasts and other cell types, such as endothelial cells and macrophages, thus strengthening our claims regarding the relevance of these signaling pathways in the progression of myocardial infarction.

It appears from this that future in vivo studies are essential in order to further validate the therapeutic potential of our findings. Specifically, we plan to perform conditional knockdown or CRISPR/ Cas9-mediated knockout of Postn or Hdac2 in animal models of MI. With these genetic manipulations, we will assess their direct effects on cardiac remodeling and fibrosis progression. Furthermore, we considered measuring important parameters such as infarct size, left ventricular function, and ejection fraction after these procedures to understand their roles in modulating cardiac function after MI. These studies will provide valuable proof-of-concept data, enhance the translational medicine credibility of our findings, and ultimately support the development of therapeutic strategies for MI fibrosis. Implementing these in vivo models will further demonstrate the relevance of our proposed targets in myocardial injury and remodeling.

## Conclusion

Based on the single-cell characterization of fibroblast subpopulations in the MI and Sham groups and the assessment of myocardial fibrosis, we propose a potential link between C1 Postn + Fibroblasts and myocardial fibrosis following MI. Therefore, C1 Postn + Fibroblasts may be more sensitive to adverse events post-MI, while other subpopulations may exhibit more suppressed sensitivity. Furthermore, the differences in stemness and metabolic regulation between C1 Postn + Fibroblasts and other subpopulations may offer key insights into strategies to prevent excessive activation of critical fibroblasts. For instance, stemness-related genes such as Cd34 and Hif1a, as well as pathways involved in oxidative phosphorylation and glutathione metabolism, should be closely monitored in future efforts to prevent post-MI myocardial fibrosis.

Regarding the interactions between fibroblasts and other cell types, potential pathways such as Cxcl12-Ackr3, Ptn-Ncl, and Mdk-Lrp1 may represent promising targets to block the primary communication between the C1 Postn + Fibroblasts and EndoCs, ECs, and macrophages, offering meaningful suggestions for therapeutic strategies to address myocardial fibrosis after MI. In addition, transcription factors such as Tead1 and Hdac2 can be verified by further experiments in the future to find their potential association with adverse outcomes such as myocardial fibrosis. Finally, in vitro validation suggests that the marker gene Postn for the C1 Postn + Fibroblasts subpopulation may play an active role in inhibiting abnormal fibroblast activation after MI.

In conclusion, these findings may open new avenues for the prevention, treatment, and progression of myocardial fibrosis following MI, with the potential to prevent its progression to HF. However, further studies and refinement of our approach are needed to fully translate these findings into clinical practice.

### Study limitations

A major limitation of this study is the small number of biological replicates, especially the MI group with only one sample (*n* = 1). This limits the universality and statistical support of the results. Therefore, we regard the analysis of the MI group as an exploratory study and emphasize the need for additional biological replicates for subsequent validation. Another limitation is the lack of experimental validation of the intercellular signaling pathways predicted by CellChat. While these computational predictions are presented, further validation via co-culture and conditioned medium experiments is needed. Additionally, the hypothesis that C1 Postn + fibroblasts signal to endocardial cells via the Cxcl12-Ackr3 axis remains untested in vivo, and the effects of modulating this pathway on infarct size or remodeling have not been explored. The hypothesis that inhibiting Tead1 and Hdac2 could attenuate fibrosis also lacks direct experimental validation. These limitations should be addressed in future studies, which will further validate these findings and explore potential therapeutic strategies. Despite these issues, the study provides a valuable foundation for future research.

## Electronic supplementary material

Below is the link to the electronic supplementary material.

Supplementary Material 1

Supplementary Material 2

Supplementary Material 3

## Data Availability

Data is provided within the manuscript. Raw and additional data related to this study can be obtained from the corresponding author (email: 71000799@sdutcm.edu.cn).
