# Supplementary material for: Single-cell RNA sequencing reveals the potential role of Postn(+) fibroblasts in promoting the progression of myocardial fibrosis after myocardial infarction
Source: Sci Rep. 2025 Jul 1;15:22390. doi: 10.1038/s41598-025-04990-6 (PMC12217889; doi:10.1038/s41598-025-04990-6)
Supplement: Supplementary file 2 — Supplementary Material 2 [file 41598_2025_4990_MOESM2_ESM.docx]

**Supplementary Material**

**Supplementary Figure1**

**A.** The UMAP plot showed that all high-quality cells were grouped into 26 distinct cell clusters.
**B.** The Bubble plot illustrated the differential gene expression across the 26 cell clusters.
**C.** The expression of fibroblast-specific marker genes was mapped across the 26 cell clusters.
**D.** UMAP plots depicted the distribution of fibroblast marker genes (Angptl7, Col1a1, Col1a2, Pdgfra, Lum, and Dcn) across the clusters.
**E.** A heatmap displayed the AUC values of the top 20 metabolic pathways in fibroblasts.
**F.** The expression patterns of oxidative phosphorylation and glutathione metabolism pathways were mapped onto the UMAP plots.
**G.** Violin plots compared the expression differences of oxidative phosphorylation and glutathione metabolism pathways across different cell types.
**H.** The Bubble plot showed the differential expression of stemness-related genes across the 10 cell types.
**I.** UMAP plots displayed the expression of stemness genes (Cd34, Twist1, Kdm5b, and Hif1a) across all cells.
**J.** Violin plots compared the expression differences of the four stemness genes (Cd34, Twist1, Kdm5b, Hif1a) across all cells.
**K.** The heatmap demonstrated the differences in heart failure and myocardial fibrosis scores across subpopulations at different cell cycle stages.
**L.** The UMAP plot showed the distribution and density differences of heart failure scores across all subpopulations.
**M.** The Violin plot compared the strength of heart failure scores across different subpopulations, showing their expression ranking.
