# Supplementary material for: Single-cell RNA sequencing reveals the potential role of Postn(+) fibroblasts in promoting the progression of myocardial fibrosis after myocardial infarction
Source: Sci Rep. 2025 Jul 1;15:22390. doi: 10.1038/s41598-025-04990-6 (PMC12217889; doi:10.1038/s41598-025-04990-6)
Supplement: Supplementary file 3 — Supplementary Material 3 [file 41598_2025_4990_MOESM3_ESM.pdf]

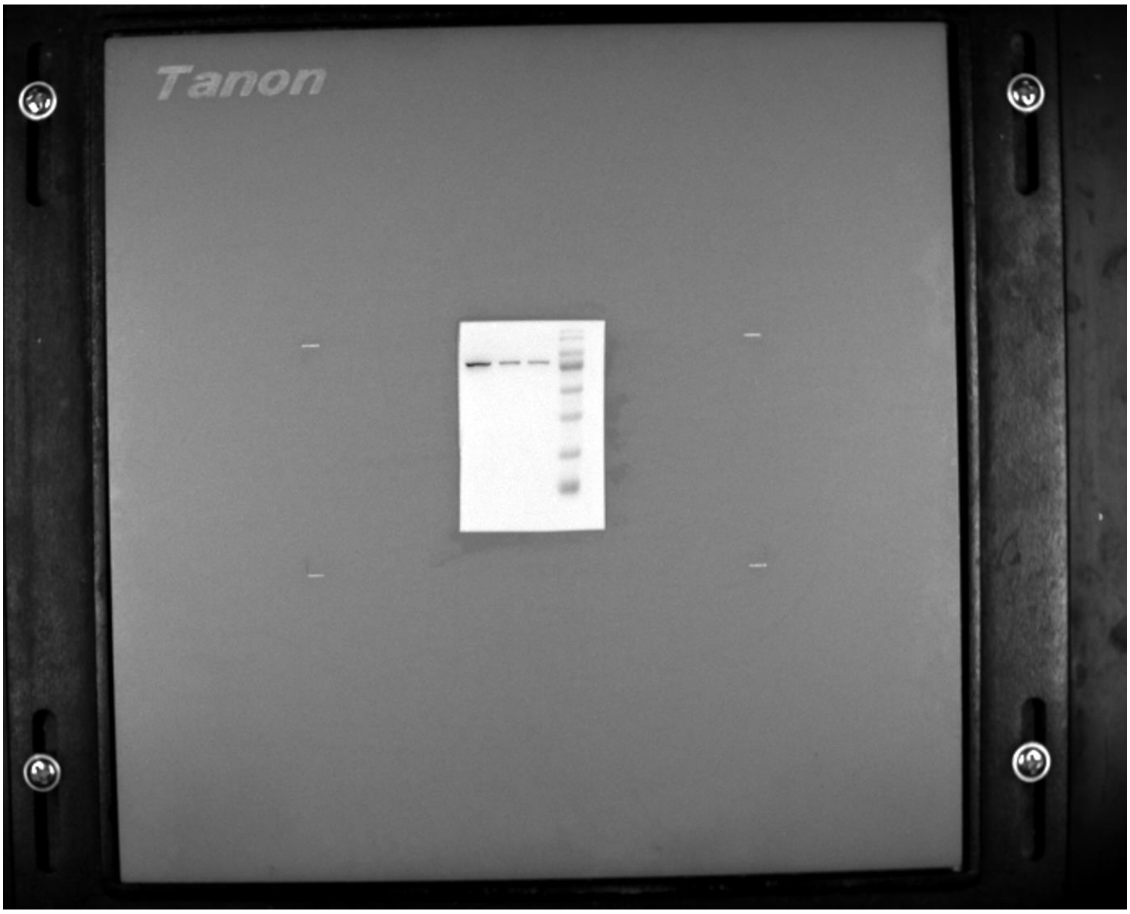

Biological repeat 1 (Postn)

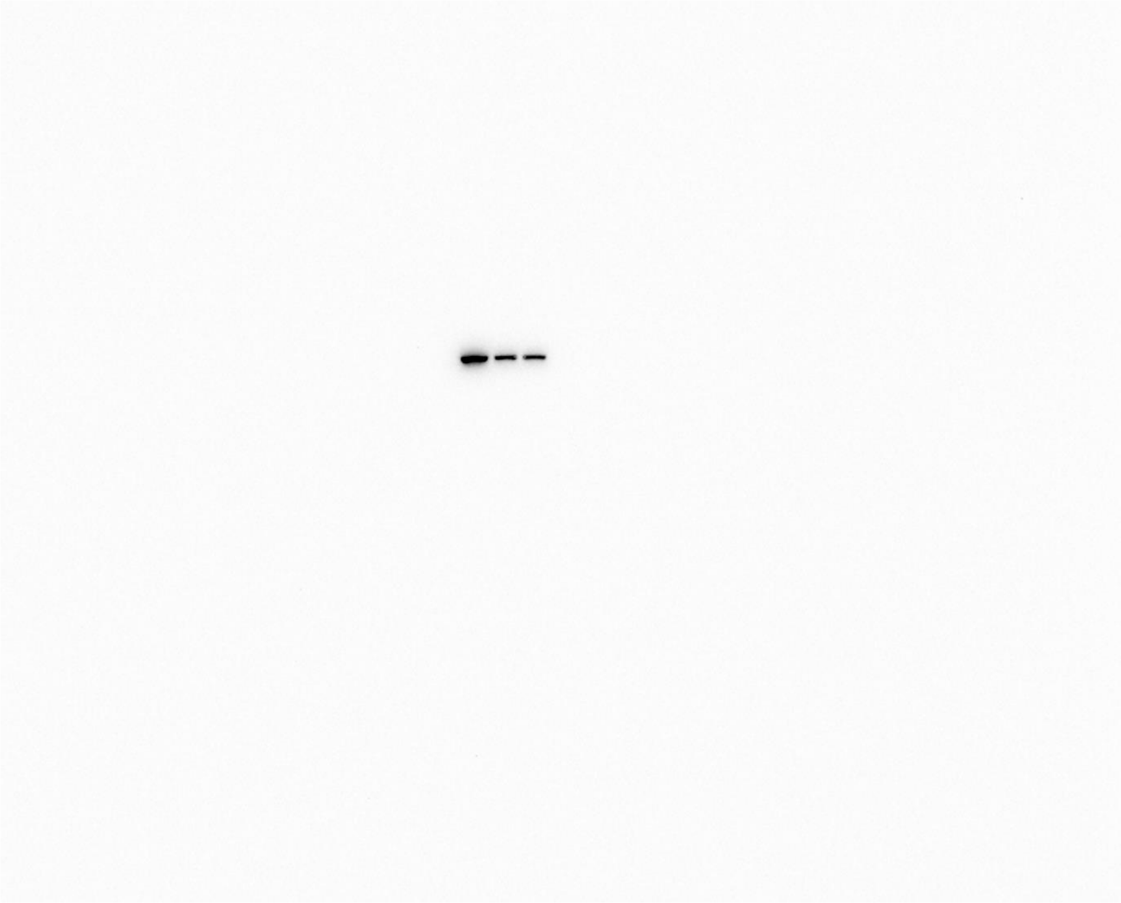

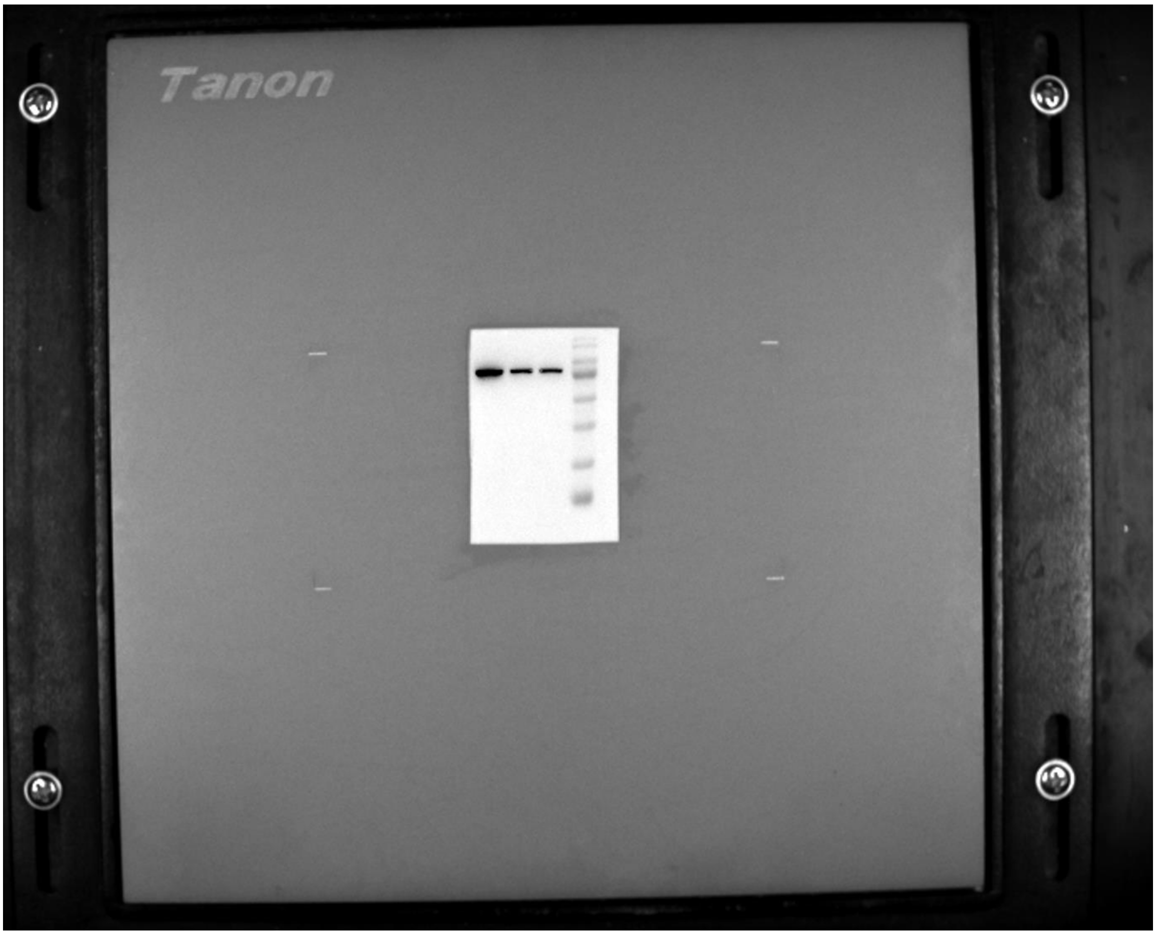

Biological repeat 2 (Postn)

The original blots used in main figure

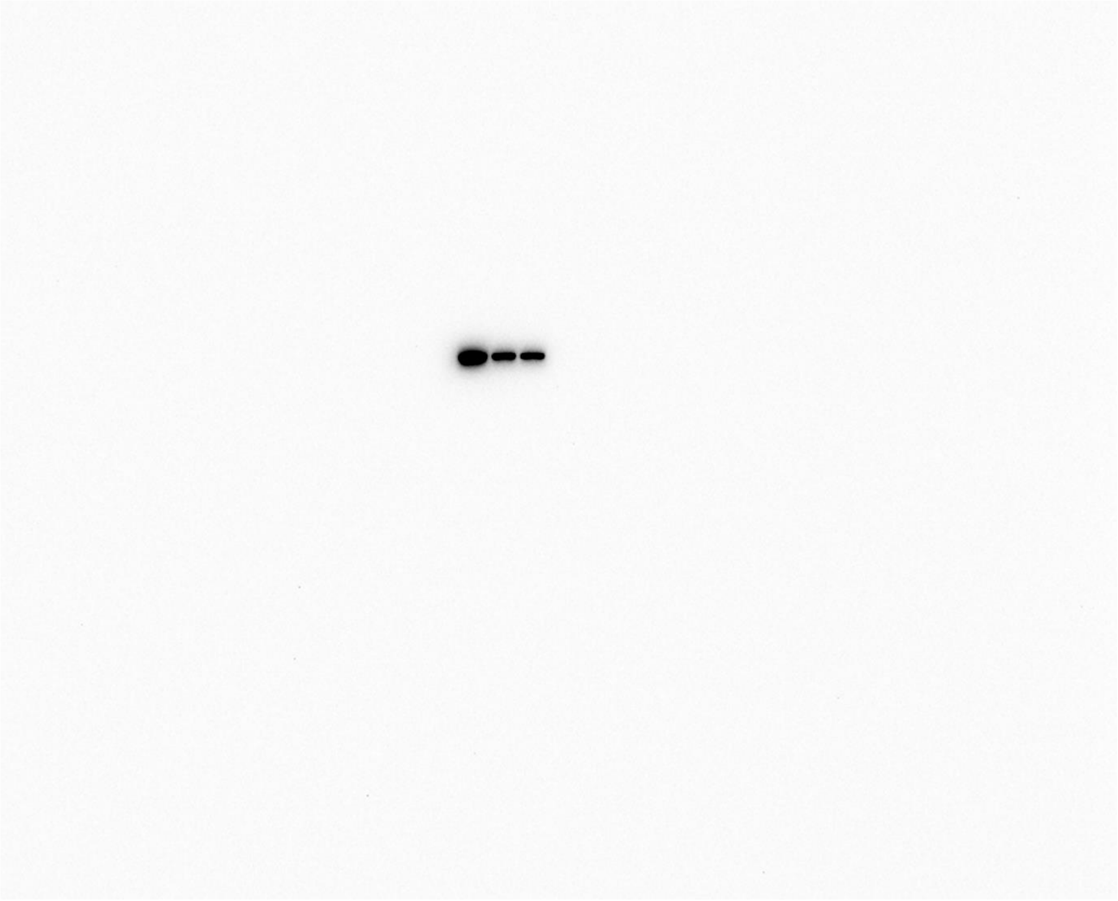

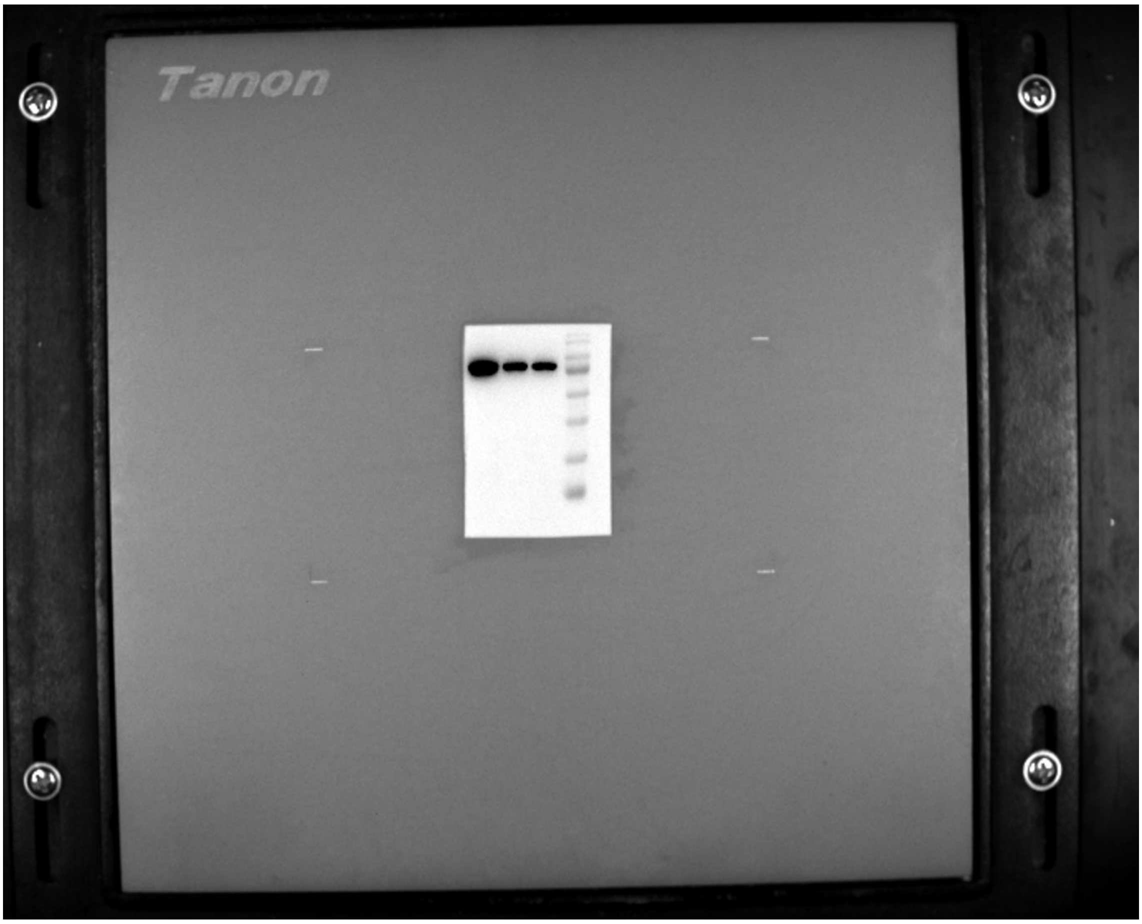

Biological repeat 3 (Postn)

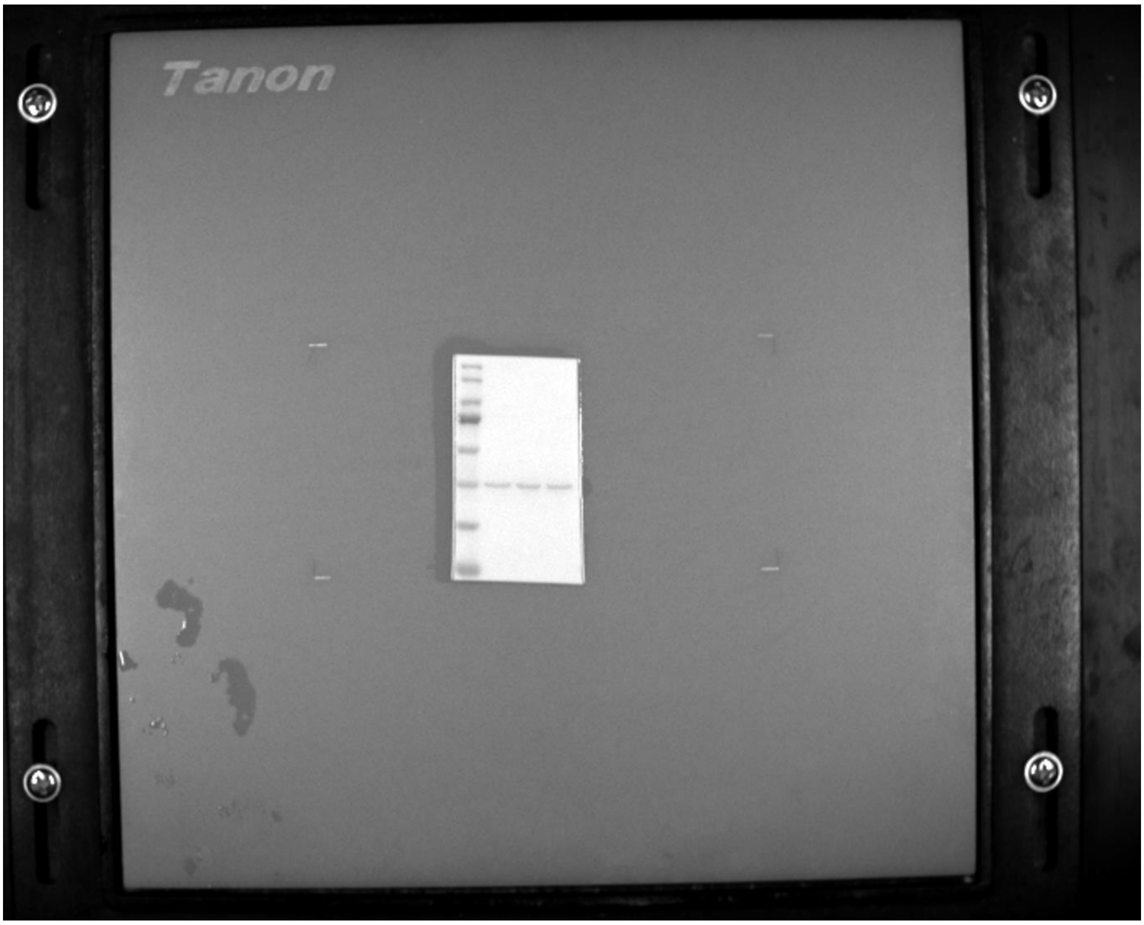

Biological repeat 1 (β-actin)

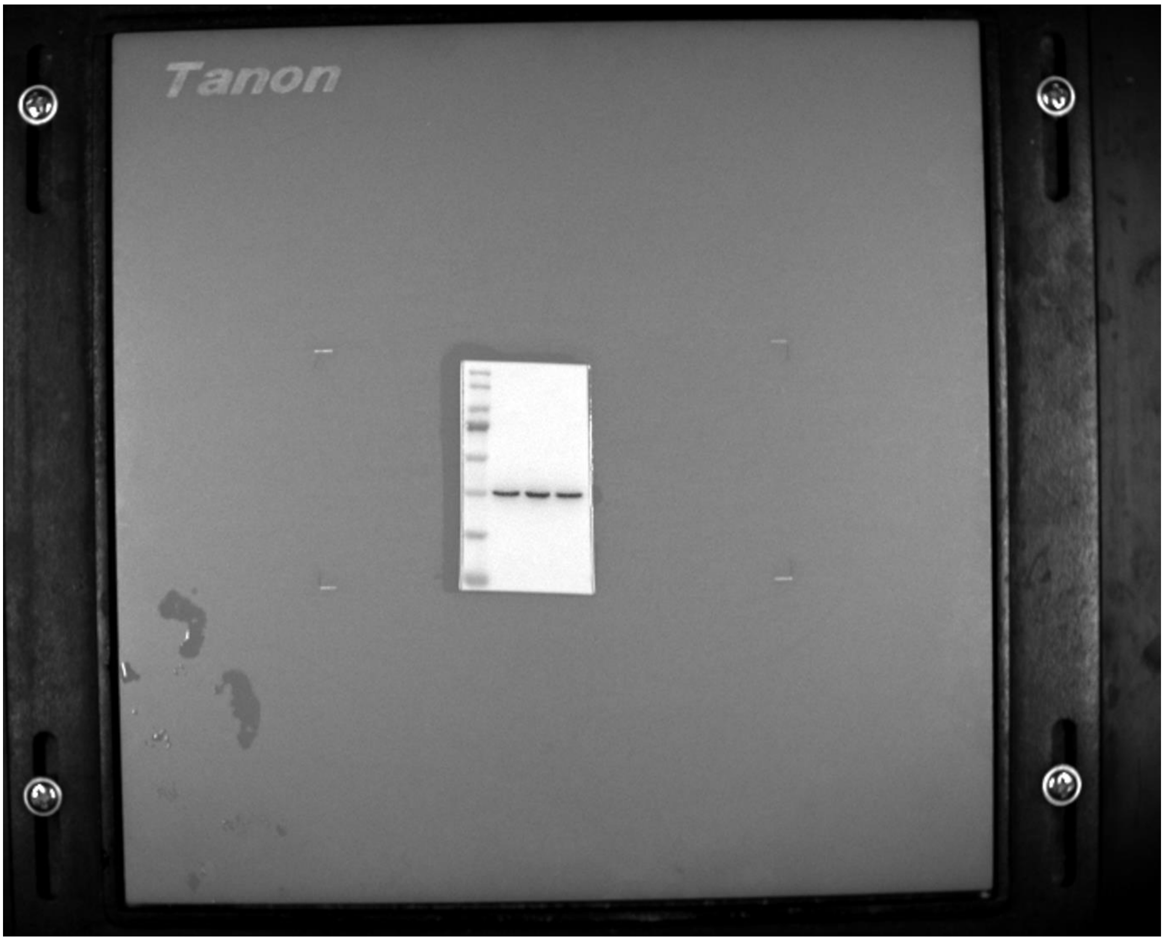

Biological repeat 2 (β-actin)

The original blots used in main figure

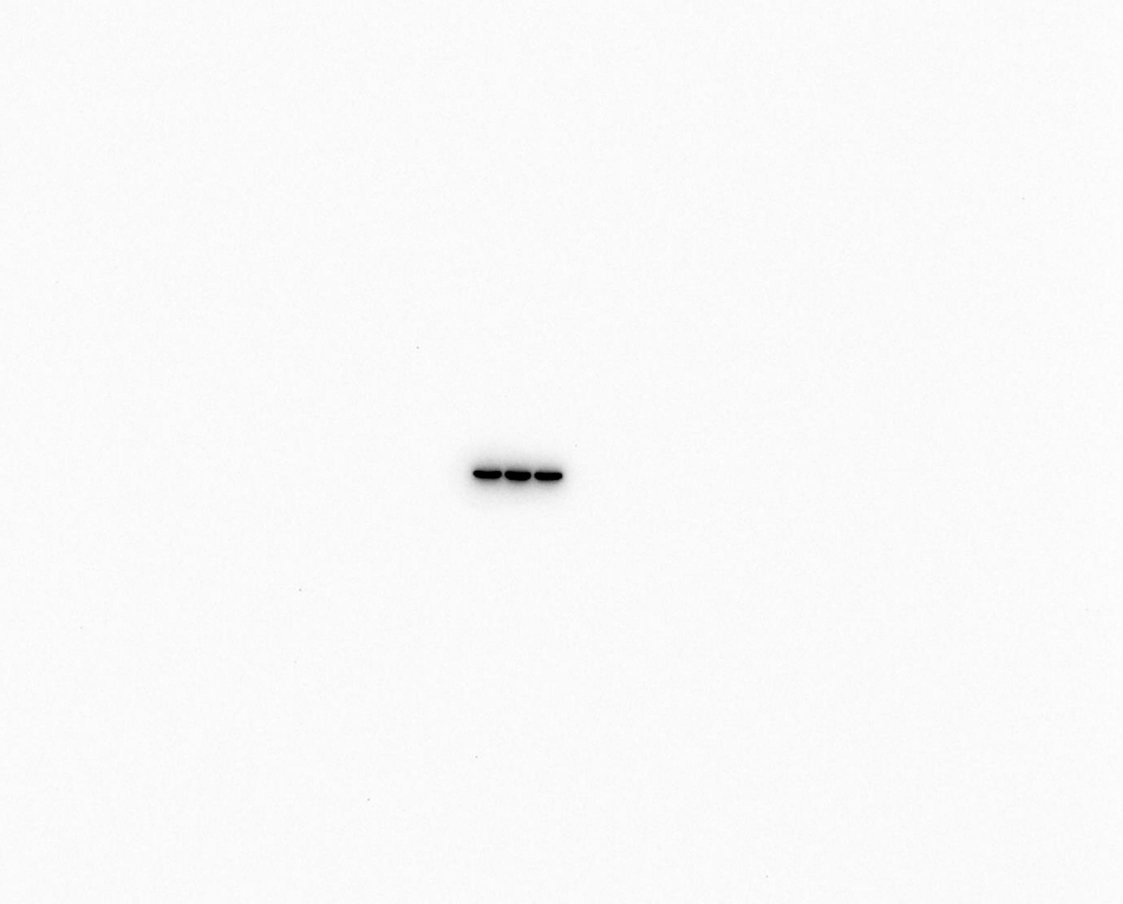

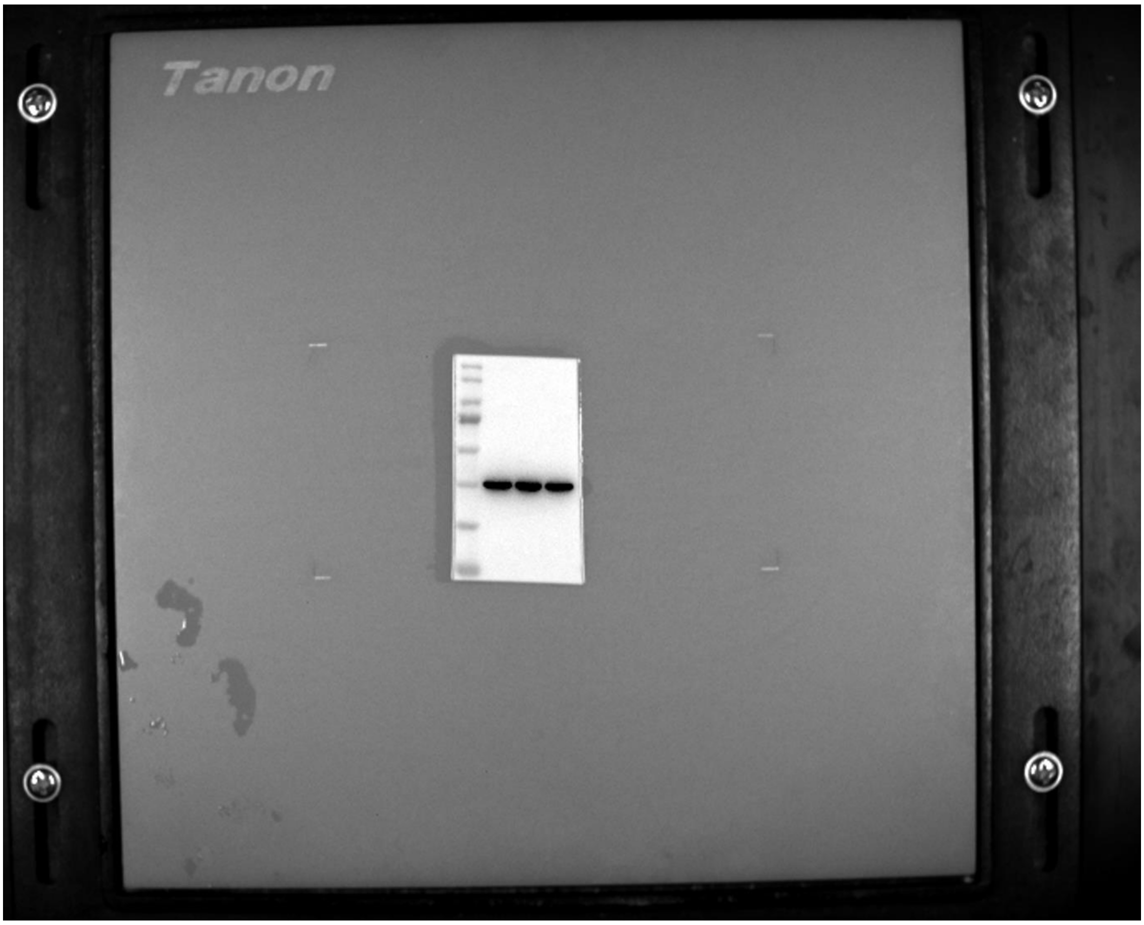

Biological repeat 3 (β-actin)
